# Rapid evaluation of 25 key sphingolipids and phosphosphingolipids in human plasma by LC-MS/MS

**DOI:** 10.1007/s00216-015-8585-6

**Published:** 2015-03-08

**Authors:** Abdul Basit, Daniele Piomelli, Andrea Armirotti

**Affiliations:** 1 Department of Drug Discovery and Development, Istituto Italiano di Tecnologia, via Morego 30, 16163 Genoa, Italy; 2 Departments of Anatomy and Neurobiology, Pharmacology and Biological Chemistry, University of California, Irvine, CA 92697 USA

**Keywords:** Ceramides, Sphingomyelins, Sphingosine-1-phosphate, Sphinganine-1-phosphate, LC-MS/MS, Human plasma

## Abstract

We report on a new, sensitive, and fast LC-MS/MS method for the simultaneous determination of 25 key sphingolipid components in human plasma, including phosphorylated sphinganine and sphingosine, in a single 9-min run. This method enables an effective and high-throughput coverage of the metabolic changes involving the sphingolipidome during physiological or pathological states. The method is based on liquid–liquid extraction followed by reversed-phase LC-MS/MS. Exogenous odd-chain lipids are used as cost-effective but reliable internal standards. The method was fully validated in surrogate matrix and naive human plasma following FDA guidelines. Sample stability and dilution integrity were also tested and verified.

**Electronic supplementary material:**

The online version of this article (doi:10.1007/s00216-015-8585-6) contains supplementary material, which is available to authorized users.

## Introduction

**Fig. 1:**
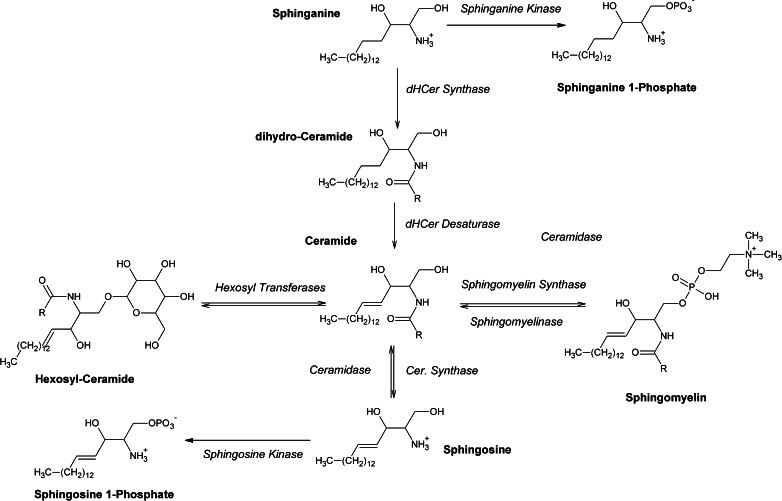
Key products and intermediates in human sphingolipidome

Sphingolipids are a class of bioactive lipid molecules characterized by a high degree of structural and functional diversity. They have been implicated in a variety of biological processes, including senescence [1], inflammation [2], and apoptosis [3, 4]. Furthermore, the plasma levels of these compounds may be altered in age-related disorders such as Alzheimer’s disease [5, 6] and mild cognitive impairment [7]. Sphingolipids also play important roles in cancer [8], diabetes [9], skin disease [10], atherosclerosis [11], and the pathogenesis of obesity [12]. Given their varied and important functions in health and disease, it is essential to be able to measure the concentrations of these biomolecules in plasma and tissues with sensitive, accurate, and reliable methods. Moreover, because of the tight metabolic interconnections among different members of the sphingolipid family, it is important to perform such measurements simultaneously on multiple rather than individual components of the family. Key products and intermediates in sphingolipid metabolism are illustrated in Fig. 1. In the de novo synthesis pathway, sphinganine is acylated to form dihydroceramides, which are converted into ceramides by the action of a desaturase. Ceramides and their corresponding sphingomyelins are metabolically connected through sphingomyelin synthase, which catalyzes the transfer of a polar choline head group onto the relatively apolar structure of ceramides. The reverse reaction, catalyzed by sphingomyelinase, represents the so-called “salvage pathway” for ceramide formation [13]. Glucosyl- and galactosyl-transferases converts ceramides into glucosylceramides and galactosylceramides, respectively. Sphingosine and sphinganine (SPH d18:1 and SPH d18:0) are phosphorylated by the action of various kinases. The signaling functions of sphingosine-1-phosphate (S1P d18:1), in particular, are currently under extensive investigation since the discovery in 1998 that S1P d18:1 is an extracellular ligand for S1PR1 receptors [14] involved in cancer development and immune responses. An important role in kidney protection after hepatic ischemia has also been recently proved for sphinganine-1-phosphate (S1P d18:0) [15]. These evidences underscore the need for analytical tools, allowing the simultaneous detection of SPH d18:1, SPH d18:0, and their corresponding phosphate analogues, along with the other major members of this lipid class like ceramides and sphingomyelins. A variety of technical approaches have been reported for the quantification of sphingolipids in biological matrices. These include thin-layer chromatography (TLC) [16], high-performance liquid chromatography (HPLC) [17], enzymatic hydrolysis followed by HPLC [18], and immunological assays [19]. In recent years, LC-MS/MS-based methods largely imposed themselves over other approaches [20–24]. However, most of these LC-MS/MS methods suffer from substantial disadvantages, which include low throughput [22], lack of full validation [10, 24], or incomplete analytical coverage (e.g., sphingomyelins) [25]. Substantial advances in lipidome investigation have also been made with shotgun-like workflows, either using combined precursor or neutral loss scan modes [26] or in scan mode using high-resolution instruments [27], but triple–quadrupole-based, targeted approaches will still represent the reference tools for clinical applications in the near future. In this field, indeed, robustness, high-throughput, and easiness of the procedures are the most important benchmarks. The simultaneous analysis of the sphingolipid metabolism (Fig. 1) is extremely challenging due to the structural heterogeneity of the sphingolipidome and the broad polarity span of its members. Differences in polarity, for example, make it difficult to group together S1P d18:1 (LogP = 3.43) and Cer d18:1/24:0 (LogP = 14.42) in the same sample preparation and the same chromatographic run. Even more challenging is to achieve their efficient separation in a short analysis time, ensuring at the same time robustness, reproducibility, and sensitivity. Several new sample preparation methods have also been established like dried blood spot extraction [28] and solid-phase extraction followed by in situ derivatization [29]. Despite these advances, the well-known Bligh-Dyer [30] total lipid extraction still remains very popular, thanks to its efficacy and ease. Here, we describe an LC-MS/MS method for the simultaneous identification and quantification of 25 key members of the sphingolipidome in human plasma, including S1P d18:1 and S1P d18:0. To our knowledge, this is the first reported LC-MS/MS method suitable for the simultaneous evaluation of the large majority of sphingolipid class representatives, and most importantly, this is the first report of an effective incorporation of sphingosine and sphinganine phosphates in a reversed-phase method focused on ceramides and sphingomyelins. The method is sensitive and robust, and it has been validated following FDA guidelines, testing for recovery, process efficiency, and matrix effects from human plasma.

## Materials and methods

### Materials

Sphingolipid standards were purchased from Avanti Polar Lipids (Alabaster, Alabama USA). Solvents and chemicals were from Sigma-Aldrich (Milan, Italy). UPLC/MS and MS/MS systems and columns were from Waters (Milford, USA).

### Human plasma samples

Healthy male and female subjects were enrolled in the MCI/AD Italian prevention project [31, 32] aimed at studying cognitive and neuropsychiatric symptoms and disorders in patients with MCI and AD at the IRCCS Santa Lucia Foundation memory clinic in Rome, Italy. The nature and purpose of the study were presented to patients and caregivers and controls, and written informed consent was obtained. The study was approved by the Ethical Committee of the Santa Lucia Foundation. Human plasma samples were collected from healthy male and female volunteers of all ages. Blood samples were taken by venipuncture in the morning after an overnight fast. Blood was collected into 10 ml tubes containing spray-coated K2EDTA (Vacutainer, Becton Dickinson, Italy). Plasma was then prepared by centrifugation of blood at 400×*g* for 15 min, then stored at −80 °C before analysis. Blood drawing and sample preparation of human plasma samples were carried out applying the best safety precautions. Plasma samples from all subjects were then pooled together and used as naïve matrix for the present study.

### Stock solution preparation

Stock solutions were prepared in methanol and chloroform mixture (1:1). An internal standard (IS) solution (200 nM Cer d18:1/17:0, 200 nM PC 23:0/23:0, 200 nM GlcCer d18:1/12:0, 250 nM SPH d17:1, 250 nM SPH d17:0, 250 nM S1P d17:1, and 250 nM S1P d17:0) was prepared by spiking the corresponding standards in the extraction solvent methanol/chloroform (2:1) added with trifluoroacetic acid (TFA) to a final 0.1 % (*v*/*v*) concentration.

### Sample preparation

Samples were extracted using a Bligh and Dyer method for lipid extraction [30]. Calibration curve (CC), quality control (QC), or test samples (50 μL) were transferred to glass vials. Liquid–liquid extraction (LLE) was carried out using a 1:2 by volume chloroform/methanol mixture (2 ml) added with TFA (final 0.1 % *v*/*v*) and spiked with the IS as described above. After mixing for 30 s with a Vortex®, chloroform (0.5 mL) and water (0.5 mL) were sequentially added, thoroughly mixing after each addition. The samples were then centrifuged for 15 min at 3500×*g* at room temperature. At the end of the process, the aqueous (upper) and organic (lower) phases were separated by a protein precipitate floating at the interface. The organic phase was then transferred to glass vials. To increase the overall recovery, the aqueous fraction was extracted again with chloroform (1 mL). The two resulting organic phases were pooled, dried under a stream of N_2_, and the residues were redissolved in methanol/chloroform (9:1, by volume; 0.1 mL). After mixing (30 s) and centrifugation (10 min at 5000×*g*, room temperature), the samples were transferred to glass vials for analyses.

### LC-MS/MS analyses

LC-MS/MS analyses of the samples were carried out on an Acquity UPLC system coupled with a Xevo TQ-MS triple-quadrupole mass spectrometer. Chromatographic separation was achieved using a BEH C18 column (2.1 × 50 mm, 1.7 micron particle size) eluted at a flow rate of 0.4 mL/min. Instruments and column were from Waters Inc. Milford, MA, USA. The mobile phase consisted of 0.1 % formic acid in acetonitrile/water (20:80 *v*/*v*) as solvent A and 0.1 % formic acid in acetonitrile/2-propanol (20:80 *v*/*v*) as solvent B. A step gradient program was developed for the best separation of all metabolites: 0.0–1.0 min 30 % B, 1.0–2.5 min 30 to 70 % B, 2.5–4.0 min 70 to 80 % B, 4.0–5.0 min 80 % B, 5.0–6.5 min 80 to 90 % B, and 6.6–7.5 min 100 % B. The column was then reconditioned to 30 % B for 1.4 min. The total run time for analysis was 9 min, and the injection volume was 3 μL. The mass spectrometer was operated in the positive ESI mode, and analytes were quantified by multiple reaction monitoring (MRM). The capillary voltage was set at 3 kV. The cone voltage was set at 25 V for all transitions, except for some SM. The complete panel of source parameters and MRM transitions are reported in the datasheet in the Electronic Supplementary Material (ESM). The source temperature was set to 120 °C. Desolvation gas and cone gas (N_2_) flow were set to 800 and 20 l/h, respectively. Desolvation temperature was set to 600 °C. Data were acquired by MassLynx software and quantified by TargetLynx software. Calibration curves were constructed by plotting the analyte to IS peak areas ratio versus the corresponding analyte concentration using weighted (1/*x* ^2^) least square regression analysis, as recommended by Gu and colleagues [33].

### Standard curves

As analytes are present endogenously in plasma, 5 % bovine serum albumin (BSA) in saline solution was used as surrogate matrix [34] for the first part of the validation process (see the “Results and discussion” for additional information). Calibration standards were prepared by spiking the analytes in the surrogate matrix. Eight-point calibration curves (1 to 1000 nM) were prepared by serial dilution into saline solution containing 5 % BSA.

### Quality control samples

Quality control samples were prepared at three different levels, as low QC (LQC), medium QC (MQC), and high QC (HQC), using the same procedure described for the standard curve to final concentrations of 19.5, 260, and 650 nM, respectively.

### Linearity, precision, and accuracy

Method linearity was tested using the eight-point calibration curve described above. The overall method performance was assessed by evaluating the accuracy and precision of back-calculated concentrations of standards and evaluating the slope, intercept, and coefficient of determination of the 1/(concentration)^2^ weighted regression line. Following FDA guidelines in this matter, acceptance criteria for the calibration curve were set to ±15 % of the nominal concentration and six out of eight calibration points had to meet the acceptance criteria. Precision and accuracy were determined by assessing the performance of quality control samples (LQC = 19.5 nM, MQC = 260 nM, and HQC = 650 nM). All the QC samples were run in triplicate. Accuracy was evaluated by calculating the percent deviation (% dev.) from nominal concentration. Precision was determined by calculating the coefficient of variation (% CV) of replicates within each batch. Acceptance criteria for precision and accuracy were defined as ≤15 % [35].

### LLOQ

The lower limit of quantification for our method was set to 1 nM for all analytes. At this value, all species showed a S/N value above 10. Six replicate 1-nM calibrators spiked in 5 % BSA were extracted and analyzed. The obtained %CV values for each analyte are reported in the ESM (supplementary datasheet).

### Sample recovery and matrix effects

Recovery and matrix effects were evaluated using the method outlined by Matuszewski et al. [36]. Three sets of samples were prepared spiking the standards at three different concentrations. Set 1 consisted of neat samples (standards spiked in reconstitution solution). Set 2 consisted of post-extraction spiked samples (blank matrix was extracted and then spiked with standards). Set 3 consisted of normal extracted samples (standards were spiked in blank matrix and then extracted). Matrix effect (ME) was calculated as (set 2/set 1) × 100. Recovery (RE) was calculated as (set 3/set 2) × 100. Human plasma matrix effect was evaluated using the post-column infusion method: A mixture of analytes diluted in 9:1 MeOH/CHCl_3_ to a final 10 μM concentration was infused post-column in the LC-MS/MS system using a tee union. Repeated injections of extracted human plasma samples were performed in the system with the aim to investigate significant decreases or increases in the analyte MRM ion currents.

### Plasma recovery

Analyte recovery from plasma was calculated using the same criteria described above: A mixture of standards was added at different concentrations in plasma (from 50 nM to 15 μM) prior or after the extraction (pre- and post-extraction spiking). The recovery was calculated as the ratio between the analyte peak area pre-spiked in plasma and the analyte peak area in post-extraction spiked samples (see “Results and discussion” for detailed information).

### Autosampler and plasma stability and dilution integrity

Plasma stability was assessed by spiking odd-chain standards in human plasma and keeping the sample under different temperature and timing conditions: short-term conditions (4 and 25 °C for 6 h) and long-term storage conditions (−20 ° C for 9 days). Sample stability in the instrument autosampler was also evaluated by re-analyzing extracted samples kept under the autosampler condition (18 h at 4 °C). All the stability studies were conducted in triplicate at 500 nM concentration. Method integrity to dilutions was also evaluated by spiking odd-chain standards in human plasma to a final 4000 nM concentration. Samples were then diluted 20-fold in blank human plasma, extracted, and analyzed.

## Results and discussion

### Chromatographic separation

**Fig. 2:**
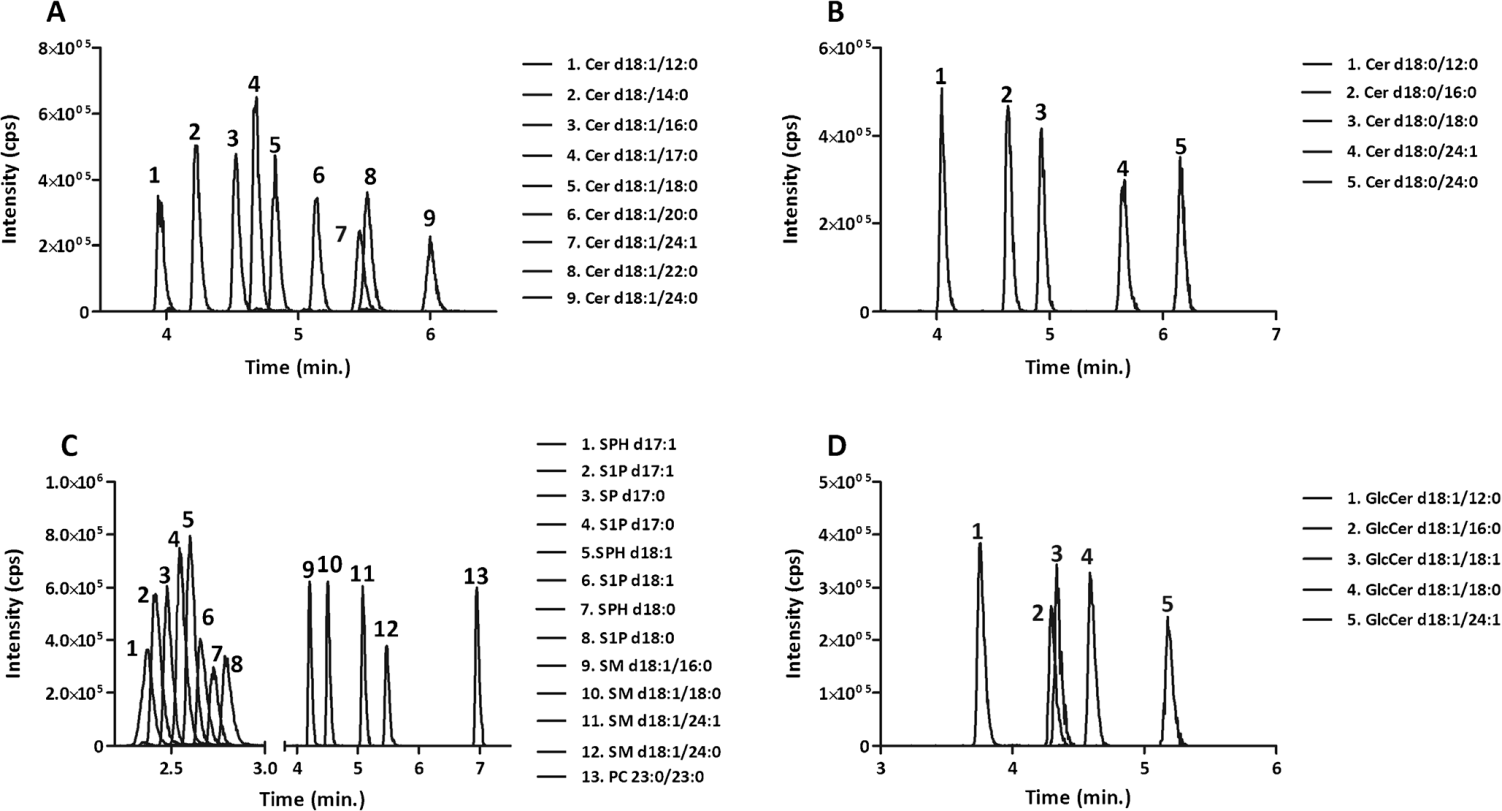
MRM chromatograms of **A** ceramides; **B** dihydroceramides; **C** sphingomyelins, sphingosines, and sphinganines; and **D** glucosylceramides spiked at 1 μM in 5 % BSA

We tested multiple combinations of stationary phases and elution conditions and eventually selected a gradient elution with an initial solvent system of low organic content, which was required to retain on the column polar compounds such as SPH d18:1 and SPH d18:0, followed by a rapid progressive increase in solvent strength, which was needed to elute from the column apolar compounds such as ceramides and dihydroceramides. The best compromise between selectivity and speed of separation was obtained using a C18 stationary phase and a high content of 2-propanol (80 %) in solvent B coupled to 20 % ACN in solvent A to reduce back pressure. Using these conditions, an adequate separation of 25 target analytes was achieved in a single 9-min-long LC-MS run (Fig. 2). In the interest of clarity, the analyte traces shown in Fig. 2 are split in four panels (A: separation of ceramides; B: dihydroceramides; C: sphingomyelins, SPH d18:1, SPH d18:0, S1P d18:1, and S1P d18:0; D: glucosylceramides).

### MS parameter optimization and choice of internal standards

**Fig. 3:**
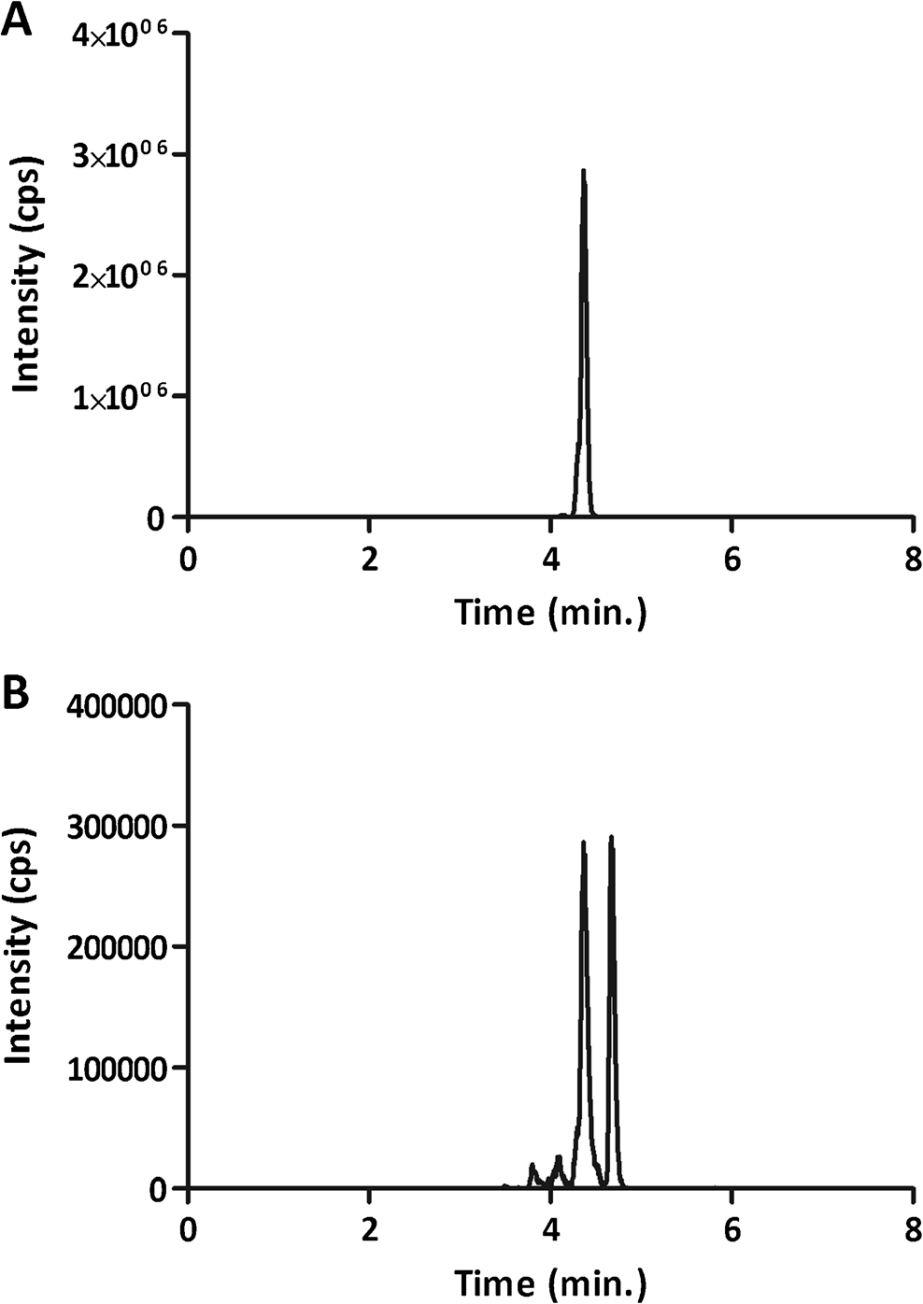
MRM trace for transition *m/z* 718->184 from standard SM d18:1/17:0 (**A**) and from extracted naïve plasma (**B**). An interfering peak in plasma shares the same transition and retention time with standard SM d18:1/17:0

To define MS parameters for optimum sensitivity, authentic standards were infused separately into the mass spectrometer. The most intense MRM transition was selected to quantify each analyte (see ESM supplementary datasheet). A total of 32 MRM transitions were included in the method. Although it is very well known that deuterated standard represents the best choice as internal standards for LC-MS/MS analysis of endogenous molecules since a very limited number of deuterated sphingolipids are commercially available, and due to the very high cost of the custom synthesis of a deuterated standard for each of the 25 sphingolipids we are tracking, we were forced to set up and validate our method using odd-chain lipids as internal standards for analytes of similar structure but with even-numbered carbon chains. The use of odd-chain standards is a very common approach for sphingolipid analysis [37], and odd-chain Cer d18:1/17:0 has already been successfully used by Haus and colleagues to determine ceramide content in plasma [9]. Since no odd-chain glucosylceramides are commercially available, we used instead the short-chain GlcCer d18:1/12:0, which was not detectable in plasma. It is important to point out that our RP method cannot distinguish between gluco- and galactosylceramides that are fully coeluting. We then chose to use glucosylceramides for the calibration curve and GlcCer d18:1/12:0 as internal standard for both gluco- and galacto- derivatives, assuming an identical instrumental response in ESI conditions, as recently confirmed by Shaner and colleagues [38]. For the same reason (full coelution and identical instrumental response) and to limit additional costs, we only used glucosylceramides in the calibration curve. Our method is then intended to evaluate the total *hexosylceramides* content in plasma. Notably, SM d18:1/17:0 did not provide a suitable internal standard for endogenous sphingomyelin species because its MRM transition (*m/z* 718->184) was found to generate an interfering peak (Fig. 3). Having excluded other options, we then concluded that SM d18:1/17:0 is endogenously present in plasma and we tested other exogenous lipid molecules potentially suitable as internal standards for sphingomyelins (see ESM Fig. S1). We tested human plasma for the MRM transitions of PC 16:0/16:0 (used by Byrdwell and Perry [39]), SM d18:1/12:0, PC 9:0/9:0, PC 15:0/15:0, and PC 17:0/17:0 (panels A to E, respectively). All these transitions generated huge ion currents (>5000–10,000 ion counts) at multiple retention times. These peaks correspond to isomeric (even/even) phosphatidylcholines like, for example, (16:0/18:0) for (17:0/17:0). We then discarded all the corresponding molecules as internal standards. The best alternative option would have been an (odd/even) phosphatidylcholine that is not commercially available. We then opted for PC 23:0/23:0 because the corresponding MRM transition from human plasma (ESM Fig. S1, panel F, black trace) generates the lowest ion current (<1500 ion counts) and does not interfere with the detection and quantification of authentic PC 23:0/23:0 (red trace).

A detailed summary of the MRM transitions and source parameters for all analytes and internal standards is reported in the ESM (supplementary datasheet). Since we are fully aware that the lack of a coeluting internal standard might represent a major problem in LC-MS/MS, we did our best to evaluate possible matrix effects using the post-column infusion method [40]. The results of this experiment are available for download as ESM. Most analytes did not show any relevant matrix effect when extracted human plasma samples were injected. Five analytes showed non-negligible matrix effects with three out of four glucosylceramides among them (16:0, 18:0, and 18:1). It is thus important to point out that, assuming identical matrix effects for galactosylceramides, the evaluation of hexosylceramides content in plasma might be affected, in the absence of commercially available deuterated standards. As expected, injections of extracted plasma caused a positive increase in the post-column infusion trace of those metabolites that are endogenously present in plasma at micromolar concentrations (sphingomyelins mostly). We also investigated the possibility of endogenous plasma phosphatidylcholines coeluting with the four sphingomyelins quantified by our method and having similar *m/z* values. For the same reasons indicated above (common 184 *m/z* fragment), this would result in interferences and inaccuracy in sphingomyelins quantification. We addressed this issue by means of high-resolution MS, a brute formula calculation software and LipidMaps database search, and we showed that, at the retention time of the four sphingomyelins targeted by our method, no phosphatidylcholines having a *m/z* value close to that selected for fragmentation (±0.5 *m/z* units) is coeluting. The results of this experiment, along with a detailed description of the experiment, are available for download as ESM.

### Sample preparation

**Fig. 4:**
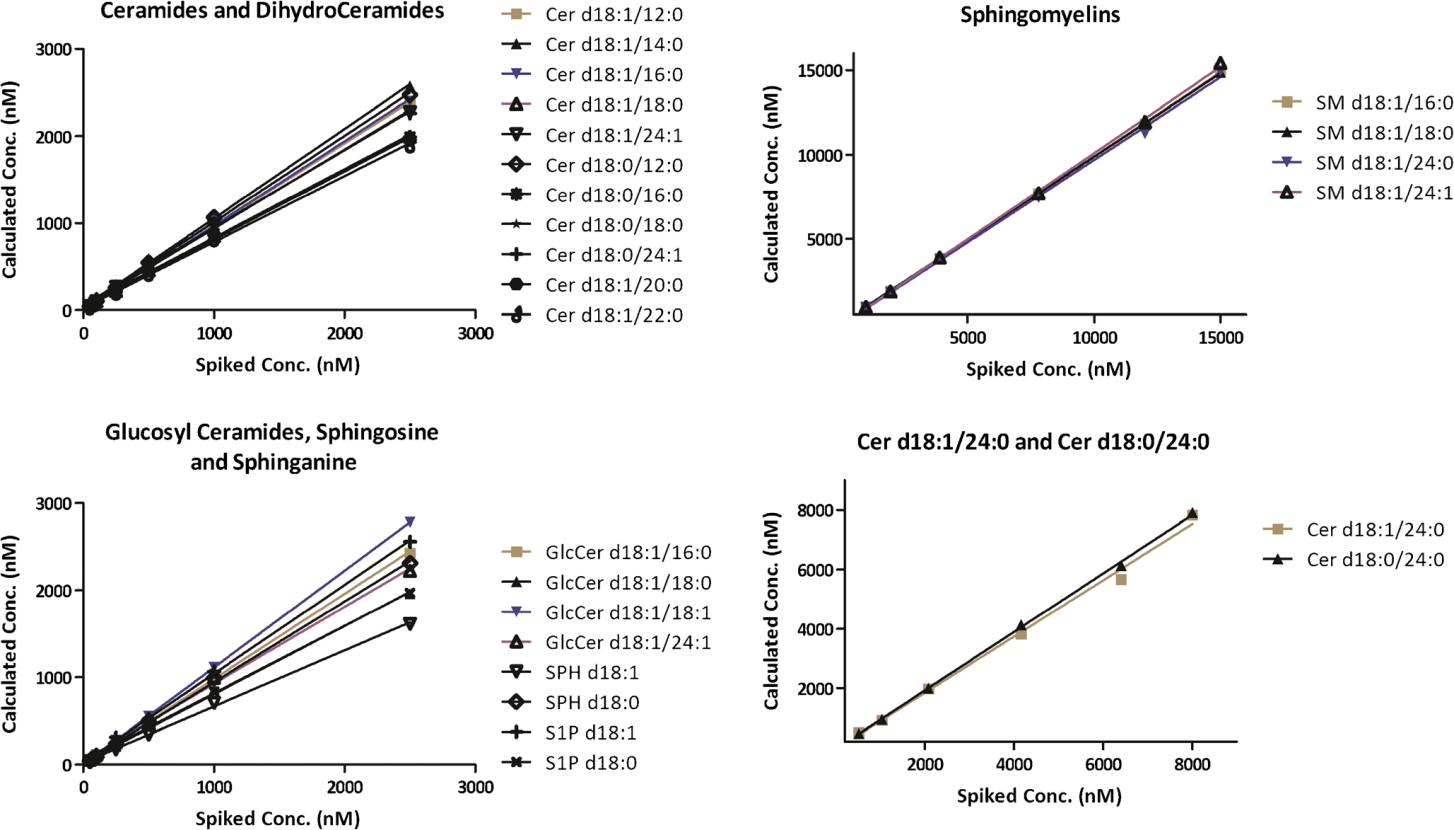
The plots shows the analyte concentration calculated after extraction and LC-MS/MS analysis versus the amount of authentic standard sphingolipids added to naïve plasma. Pre-spiking naïve plasma levels were considered as background levels and subtracted

Sphingolipids are naturally present in plasma, where they can reach relatively high concentrations (10–20 μM for certain species). This hindered the use of plasma as blank analytical matrix and interfered with our ability to validate the method. Since depletion strategies (for example, using charcoal [41, 42]) did not perform well in our hands, we switched to a surrogate plasma-like matrix constituted of a saline solution containing diluted, lipid-free, bovine serum albumin (BSA, 5 g/0.1 L), which has been widely used in the past to mimic plasma in bioanalysis [43–45]. We fully validated the method using this artificial matrix. Linearity, accuracy, precision, matrix effect, and recovery were evaluated for each analyte. Full validation data are available for download as ESM. Next, we determined linearity and recovery in human plasma samples. We first used odd-numbered chain internal standards and GlcCer(d18:1/12:0) to test process efficiency: Different combinations of analytes to extraction solution (ES) volume ratios were examined. We found that while a 1:6 ratio was adequate for 5 % BSA, a 1:40 ratio was required to ensure a good recovery from plasma. We then focused on S1P(d18:1) and S1P(d18:0), where the hydrophilic/hydrophobic dichotomy of sphingolipids is particularly relevant. At neutral pH, these molecules have a positively charged amino group and a negatively charged phosphate group, along with a 14 carbon long aliphatic chain. Without changes in the pH, the extraction efficiency of S1P(d18:1) and S1P(d18:0) from plasma is rather low (approximately 20 % for both) with a large fraction (>75 %) of analyte that remains in the upper aqueous phase. We then added TFA to the extraction solvent (0.1 % in volume) to force the protonation of both the phosphate group and the amino group, reducing the span in the protonation states. The trifluoroacetate anion also served as ion-pairing agent. TFA was chosen due to its volatility. This expedient increased the recovery of S1P(d18:1) and S1P(d18:0) to more than 80 % and had no significant effect on the recovery of other species. With this information in hand, to obtain a more complete picture of the extraction efficiencies from human plasma for all analytes, we prepared a set of human plasma samples spiked with higher-than-background concentrations of standards (from 50 nM to 15 μM). Plasma samples were also extracted, analyzed, and considered as background level. We then plotted the sphingolipids concentration against the total amount of standards added. The amount of sphingolipids naturally present in the naïve samples was subtracted from each point of the graph. Results are reported in Fig. 4. As shown in the graphs, the method yields linear recoveries when applied to human plasma samples. Furthermore, in order to calculate the plasma recovery values for individual analytes, each point was prepared using both pre-extraction and post-extraction spiking for each analyte. The recovery was then calculated from the ratio between pre-extraction and post-extraction spiking. Table 1 shows the averaged recovery values from surrogate matrix and human plasma for each sphingolipid class. The full data panel, with data on individual species, may be found in the ESM (Supplementary Datasheet).

**Table 1:** Recovery values by sphingolipid class (mean ± standard deviation) from 5 % BSA and from human plasma samples

Sphingolipids	BSA 5 %	Human plasma
Ceramides	89.66 ± 2.40	95.74 ± 7.21
Dihydroceramides	91.53 ± 1.70	96.89 ± 5.03
Sphingomyelins	82.97 ± 0.79	97.82 ± 1.36
Glucosylceramides	89.93 ± 2.54	100.93 ± 7.15
SPH(d18:1)	116.11 ± 2.94	71.93 ± 4.19
SPH(d18:0)	116.86 ± 1.16	93.69 ± 4.90
S1P(d18:1)	76.93 ± 2.45	105.01 ± 14.08
S1P(d18:0)	75.89 ± 0.71	86.64 ± 4.21

### Linearity, precision, accuracy, carryover, stability, and dilution integrity

Our method showed a very good linearity over a 10^3^-fold range (1–1000 nM, eight calibration points) with an *r* ^2^ value of 0.9895–0.9973 using 5 % BSA as matrix and 0.9947–1.0000 using human plasma as matrix. A signal-to-noise ratio (S/N) of ≥10 was considered as lowest limit of quantification to determine method sensitivity, although for most analytes the S/N at 1 nM was higher than 10. For this reason, lower limit of quantification (LLOQ) from 5% BSA was set to 1 nM. At this concentration, all analytes showed a %CV below 20 % (see ESM, supplementary datasheet). Precision and accuracy were also determined in 5 % BSA, assessing the performance of three quality control samples (LQC = 19.5 nM, MQC = 260 nM, and HQC = 650 nM). Accuracy was evaluated by calculating the percent deviation from nominal concentrations. Precision was determined by calculating the coefficient of variation of replicates within each batch. All the QC samples were run in triplicate. Acceptance criteria for precision and accuracy were defined as ≤15 % [35]. Intra- and inter-assay precision and accuracy were also tested by analyzing the same QC samples. The full dataset is reported in the ESM (supplementary datasheet). Furthermore, in order to evaluate carryover, a blank sample was injected immediately after the highest standard. Since some carryover was observed with the usual water/methanol/ACN/2-propanol needle washing system, we used acetonitrile/water (1:1) containing 10 % acetone as needle wash solvent. With this expedient, no significant carryover was observed for any of the analytes (less than 0.5 %). Analyte stability evaluation was also included in the experimental layout: Odd-chain standards were spiked in naïve human plasma to a final 500 nM concentration (triplicate samples) and tested for stability in both short-term (4 and 25 °C for 6 h) and long-term conditions (9 days at −20 °C). The stability of extracted samples in the instrument autosampler conditions (4 °C for 6 h) was also evaluated. Analytes are stables in all the abovementioned conditions (our acceptance criteria were set to ±15 % of nominal concentration). Since sphingolipids are present in human plasma at very different concentrations (from low nM to high μM), we also tested the dilution integrity of our method in order to be able to confidently quantify samples above the upper limit of the calibration curve. We then spiked odd-chain standards in naïve human plasma at a final 4 μM concentration, and we diluted the samples 20-fold using naïve human plasma. After sample extraction and analysis, analyte concentrations were calculated using the appropriate multiplication factor. Results demonstrate that our method is tolerant to 20-fold dilution since the calculated concentrations were within the acceptance criteria (precision 0.16–4.82 %, accuracy 83.04–94.25 %). Data on stability and dilution integrity are reported in the ESM (supplementary datasheet). All the sphingolipids that were detected and quantified with our method in naïve human plasma are reported in the supplementary datasheet in the ESM.

## Conclusions

In the present report, we describe a new LC-MS/MS method that allows for the rapid identification and quantification of 25 key sphingolipid species in human plasma, including S1P d18:1 and S1P d18:0. We proved that a LogP span of more than 10 units (from 3.43 of S1P d18:1 to 14.42 of Cer d18:1/24:0 can be efficiently explored in a single sample preparation and LC-MS run. While other validated methods for the LC-MS/MS quantification of sphingolipids are available in the literature, none of them offers a comparable level of analyte coverage, rapidity, and validation. For example, Bui et al. [25] describe a 5-min separation but do not cover sphingomyelins; Kasumov and colleagues [22] describe a separation of seven species in 15 min. Other methods, though validated, offer an even more limited analyte coverage: Cer d18:1/22:0 and Cer d18:1/24:0 [23] or seven species by nano-LC/MS [21]. The method we propose here is sensitive, linear, robust, and very rapid (9 min per run). Although it suffers from some matrix effect that cannot be fully compensated by the use of odd-chain internal standards, we managed to evaluate this effect and control it, achieving very promising results from human plasma. Future implementations of this method with custom-made deuterated internal standards will hopefully improve the overall method performance, making it suitable for clinical routine. The huge costs for custom synthesis will surely be compensated by the final outcome on health-care systems since sphingolipids and phosphorylated sphingolipids have been proposed as biomarkers for various pathological conditions. The potential of the method presented here is manifested as it permits a complete and reliable analysis of the full sphingolipid core metabolism in less than 10 min. Furthermore, in addition to the whole sphingolipids panel, the possibility to track S1P d18:1 and S1P d18:0 in plasma could be of great importance for clinical research on cancer and immune system disorders.

## Electronic supplementary material

Below is the link to the electronic supplementary material.

ESM 1 (PDF 5384 kb)

## References

[1] Webb LM, Arnholt AT, Venable ME (2010). Phospholipase D modulation by ceramide in senescence. Mol Cell Biochem.

[2] Teichgräber V, Ulrich M, Endlich N, Riethmüller J, Wilker B, De Oliveira-Munding CC, van Heeckeren AM, Barr ML, von Kürthy G, Schmid KW, Weller M, Tümmler B, Lang F, Grassme H, Döring G, Gulbins E (2008). Ceramide accumulation mediates inflammation, cell death and infection susceptibility in cystic fibrosis. Nat Med.

[3] Posse de Chaves EI (2006). Sphingolipids in apoptosis, survival and regeneration in the nervous system. Biochim Biophys Acta.

[4] Young MM, Kester M, Wang HG (2013). Sphingolipids: regulators of crosstalk between apoptosis and autophagy. J Lipid Res.

[5] He X, Huang Y, Li B, Gong CX, Schuchman EH (2010). Deregulation of sphingolipid metabolism in Alzheimer’s disease. Neurobiol Aging.

[6] Mielke MM, Haughey NJ, Bandaru VV, Weinberg DD, Darby E, Zaidi N, Pavlik V, Doody RS, Lyketsos CG (2011). Plasma sphingomyelins are associated with cognitive progression in Alzheimer’s disease. J Alzheimers Dis.

[7] Mielke MM, Haughey NJ, Ratnam Bandaru VV, Schech S, Carrick R, Carlson MC, Mori S, Miller MI, Ceritoglu C, Brown T, Albert M, Lyketsos CG (2010). Plasma ceramides are altered in mild cognitive impairment and predict cognitive decline and hippocampal volume loss. Alzheimers Dement.

[8] Bieberich E (2008). Ceramide signaling in cancer and stem cells. Futur Lipidol.

[9] Haus JM, Kashyap SR, Kasumov T, Zhang R, Kelly KR, Defronzo RA, Kirwan JP (2009). Plasma ceramides are elevated in obese subjects with type 2 diabetes and correlate with the severity of insulin resistance. Diabetes.

[10] Farwanah H, Pierstorff B, Schmelzer CE, Raith K, Neubert RH, Kolter T, Sandhoff K (2007). Separation and mass spectrometric characterization of covalently bound skin ceramides using LC/APCI-MS and Nano-ESI-MS/MS. J Chromatogr B Anal Technol Biomed Life Sci.

[11] Bismuth J, Lin P, Yao Q, Chen C (2008). Ceramide: a common pathway for atherosclerosis?. Atherosclerosis.

[12] Samad F, Hester KD, Yang G, Hannun YA, Bielawski J (2006). Altered adipose and plasma sphingolipid metabolism in obesity: a potential mechanism for cardiovascular and metabolic risk. Diabetes.

[13] Kitatani K, Idkowiak-Baldys J, Hannun YA (2008). The sphingolipid salvage pathway in ceramide metabolism and signaling. Cell Signal.

[14] Hanson MA, Roth CB, Jo E, Griffith MT, Scott FL, Reinhart G, Desale H, Clemons B, Cahalan SM, Schuerer SC, Sanna MG, Han GW, Kuhn P, Rosen H, Stevens RC (2012). Crystal structure of a lipid G protein-coupled receptor. Science.

[15] Park SW, Kim M, Chen SW, Brown KM, D’Agati VD, Lee HT (2010). Sphinganine-1-phosphate protects kidney and liver after hepatic ischemia and reperfusion in mice through S1P1 receptor activation. Lab Investig.

[16] Bruce CR, Thrush AB, Mertz VA, Bezaire V, Chabowski A, Heigenhauser GJ, Dyck DJ (2006). Endurance training in obese humans improves glucose tolerance and mitochondrial fatty acid oxidation and alters muscle lipid content. Am J Physiol Endocrinol Metab.

[17] Yano M, Kishida E, Muneyuki Y, Masuzawa Y (1998). Quantitative analysis of ceramide molecular species by high performance liquid chromatography. J Lipid Res.

[18] Lee S, Lee YS, Choi KM, Yoo KS, Sin DM, Kim W, Lee YM, Hong JT, Yun YP, Yoo HS (2012). Quantitative analysis of sphingomyelin by high-performance liquid chromatography after enzymatic hydrolysis. Evid Based Complement Alternat Med.

[19] Cowart LA, Szulc Z, Bielawska A, Hannun YA (2002). Structural determinants of sphingolipid recognition by commercially available anti-ceramide antibodies. J Lipid Res.

[20] Liou YB, Sheu MT, Liu DZ, Lin SY, Ho HO (2010). Quantitation of ceramides in nude mouse skin by normal-phase liquid chromatography and atmospheric pressure chemical ionization mass spectrometry. Anal Biochem.

[21] Thomas D, Eberle M, Schiffmann S, Zhang DD, Geisslinger G, Ferreiros N (2013). Nano-LC-MS/MS for the quantitation of ceramides in mice cerebrospinal fluid using minimal sample volume. Talanta.

[22] Kasumov T, Huang H, Chung YM, Zhang R, McCullough AJ, Kirwan JP (2010). Quantification of ceramide species in biological samples by liquid chromatography electrospray ionization tandem mass spectrometry. Anal Biochem.

[23] Jiang H, Hsu FF, Farmer MS, Peterson LR, Schaffer JE, Ory DS, Jiang X (2013). Development and validation of LC-MS/MS method for determination of very long acyl chain (C22:0 and C24:0) ceramides in human plasma. Anal Bioanal Chem.

[24] Farwanah H, Kolter T, Sandhoff K (2011). Mass spectrometric analysis of neutral sphingolipids: methods, applications, and limitations. Biochim Biophys Acta.

[25] Bui HH, Leohr JK, Kuo MS (2012). Analysis of sphingolipids in extracted human plasma using liquid chromatography electrospray ionization tandem mass spectrometry. Anal Biochem.

[26] Eisinger K, Krautbauer S, Hebel T, Schmitz G, Aslanidis C, Liebisch G, Buechler C (2014). Lipidomic analysis of the liver from high-fat diet induced obese mice identifies changes in multiple lipid classes. Exp Mol Pathol.

[27] Schuhmann K, Almeida R, Baumert M, Herzog R, Bornstein SR, Shevchenko A (2012). Shotgun lipidomics on a LTQ Orbitrap mass spectrometer by successive switching between acquisition polarity modes. J Mass Spectrom.

[28] Legnini E, Orsini JJ, Muhl A, Johnson B, Dajnoki A, Bodamer OA (2012). Analysis of acid sphingomyelinase activity in dried blood spots using tandem mass spectrometry. Ann Lab Med.

[29] Sanchez BA, Capote FP, Luque de Castro MD (2011). Targeted analysis of sphingoid precursors in human biofluids by solid-phase extraction with in situ derivatization prior to mu-LC-LIF determination. Anal Bioanal Chem.

[30] Bligh EG, Dyer WJ (1959). A rapid method of total lipid extraction and purification. Can J Biochem Physiol.

[31] Palmer K, Di Iulio F, Varsi AE, Gianni W, Sancesario G, Caltagirone C, Spalletta G (2010). Neuropsychiatric predictors of progression from amnestic-mild cognitive impairment to Alzheimer’s disease: the role of depression and apathy. J Alzheimers Dis.

[32] Di Iulio F, Palmer K, Blundo C, Casini AR, Gianni W, Caltagirone C, Spalletta G (2010). Occurrence of neuropsychiatric symptoms and psychiatric disorders in mild Alzheimer’s disease and mild cognitive impairment subtypes. Int Psychogeriatr.

[33] Gu H, Liu G, Wang J, Aubry AF, Arnold ME (2014). Selecting the correct weighting factors for linear and quadratic calibration curves with least-squares regression algorithm in bioanalytical LC-MS/MS assays and impacts of using incorrect weighting factors on curve stability, data quality, and assay performance. Anal Chem.

[34] van de Merbel NC (2008). Quantitative determination of endogenous compounds in biological samples using chromatographic techniques. Trends Anal Chem.

[35] Booth B, Arnold ME, DeSilva B, Amaravadi L, Dudal S, Fluhler E, Gorovits B, Haidar SH, Kadavil J, Lowes S, Nicholson R, Rock M, Skelly M, Stevenson L, Subramaniam S, Weiner R, Woolf E. (2014) Workshop report: crystal city V-Quantitative bioanalytical method validation and implementation: the 2013 revised FDA guidance. AAPS journal. doi:10.1208/s12248-014-9696-2 10.1208/s12248-014-9696-2 PMC4365089 25549614

[36] Matuszewski BK, Constanzer ML, Chavez-Eng CM (2003). Strategies for the assessment of matrix effect in quantitative bioanalytical methods based on HPLC-MS/MS. Anal Chem.

[37] Lan T, Bi H, Liu W, Xie X, Xu S, Huang H (2011). Simultaneous determination of sphingosine and sphingosine 1-phosphate in biological samples by liquid chromatography-tandem mass spectrometry. J Chromatogr B Anal Technol Biomed Life Sci.

[38] Shaner RL, Allegood JC, Park H, Wang E, Kelly S, Haynes CA, Sullards MC, Merrill AH (2009). Quantitative analysis of sphingolipids for lipidomics using triple quadrupole and quadrupole linear ion trap mass spectrometers. J Lipid Res.

[39] Byrdwell WC, Perry RH (2006). Liquid chromatography with dual parallel mass spectrometry and (31)P nuclear magnetic resonance spectroscopy for analysis of sphingomyelin and dihydrosphingomyelin I. Bovine brain and chicken egg yolk. J Chromatogr A.

[40] Geis-Asteggiante L, Lehotay SJ, Lightfield AR, Dutko T, Ng C, Bluhm L (2012). Ruggedness testing and validation of a practical analytical method for >100 veterinary drug residues in bovine muscle by ultrahigh performance liquid chromatography-tandem mass spectrometry. J Chromatogr A.

[41] Evseenko D, Latour B, Richardson W, Corselli M, Sahaghian A, Cardinal S, Zhu Y, Chan R, Dunn B, Crooks GM (2013). Lysophosphatidic acid mediates myeloid differentiation within the human bone marrow microenvironment. PLoS One.

[42] Chen RF (1967). Removal of fatty acids from serum albumin by charcoal treatment. J Biol Chem.

[43] Jones BR, Schultz GA, Eckstein JA, Ackermann BL (2012). Surrogate matrix and surrogate analyte approaches for definitive quantitation of endogenous biomolecules. Bioanalysis.

[44] Chen S, Wu JT, Huang R (2012). Evaluation of surrogate matrices for standard curve preparation in tissue bioanalysis. Bioanalysis.

[45] Bowen CL, Kehler J, Evans CA (2010). Development and validation of a sensitive and selective UHPLC-MS/MS method for simultaneous determination of both free and total eicosapentaeonic acid and docosahexenoic acid in human plasma. J Chromatogr B Anal Technol Biomed Life Sci.

