# Supplementary material for: Rapid evaluation of 25 key sphingolipids and phosphosphingolipids in human plasma by LC-MS/MS
Source: Anal Bioanal Chem. 2015 Mar 8;407(17):5189–98. doi: 10.1007/s00216-015-8585-6 (PMC4471391; doi:10.1007/s00216-015-8585-6)
Supplement: Supplementary file 1 — (PDF 5384 kb) [file 216_2015_8585_MOESM1_ESM.pdf]

Analytical and Bioanalytical Chemistry

Electronic Supplementary Material

Rapid evaluation of 25 key sphingolipids and phosphosphingolipids in human plasma by LC-MS/MS

Abdul Basit, Daniele Piomelli, Andrea Armirotti

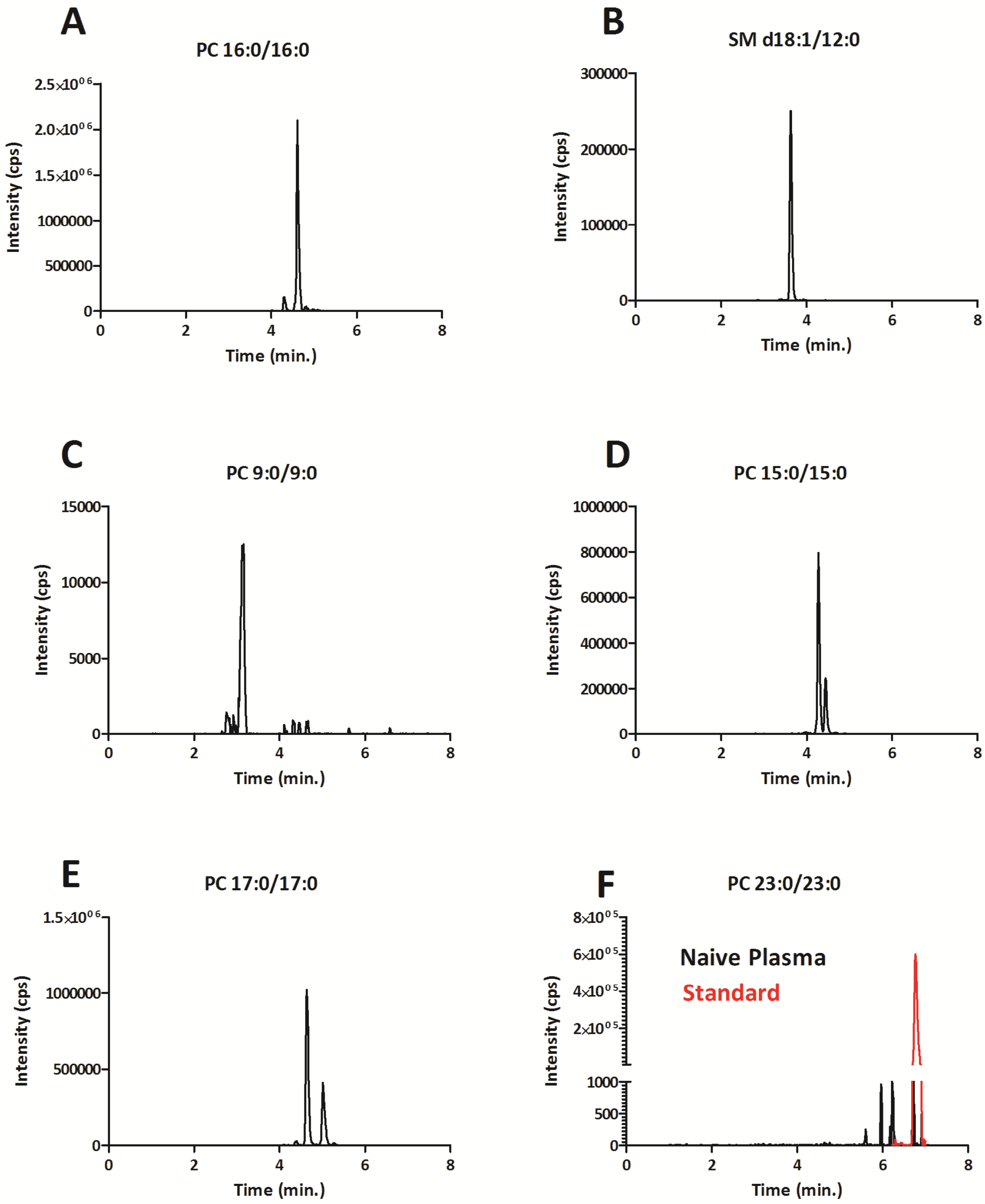

Fig. S1 MRM traces for the different analytes tested as Internal standard from extracted naïve plasma. A: PC 16:0/16:0, B: SM d18:1/12:0, C to F: PC 9:0/9:0, PC 15:0/15:0, PC 17:0/17:0 and PC 23:0/23:0

Calibration Curve data in BSA

Sphingolipids	Spike 1 nM	Spike 2 nM	Spike 5 nM	Spike 10 nM	Spike 50 nM	Spike 100 nM	Spike 500 nM	Spike 1000 nM	r2 (Mean ± SD)	Slope (Mean ± SD)	Intercept (Mean ± SD)
Ceramide (d18:1/12:0)	0.99 ± 0.06	2.01 ± 0.23	5.18 ± 0.36	10.17 ± 0.37	51.66 ± 0.39	98.42 ± 1.56	477.82 ± 19.30	989.01 ± 6.67	0.9959 ± 0.0012	1.1268 ± 0.065	0.2931 ± 0.335
Ceramide (d18:1/14:0)	0.95 ± 0.05	2.12 ± 0.20	5.34 ± 0.09	10.83 ± 0.19	51.14 ± 0.64	100.58 ± 4.16	454.81 ± 9.44	907.16 ± 13.11	0.9914 ± 0.003	1.225 ± 0.061	-0.1527 ± 0.0575
Ceramide (d18:1/16:0)	1 ± 0.06	1.92 ± 0.25	5.23 ± 0.28	10.92 ± 0.62	52.09 ± 0.58	101.58 ± 2.66	456.79 ± 19.54	932.68 ± 42.93	0.9915 ± 0.003	1.1049 ± 0.0672	0.5207 ± 0.5972
Ceramide (d18:1/18:0)	0.95 ± 0.02	2.16 ± 0.08	5.1 ± 0.30	10.41 ± 0.66	54.04 ± 0.96	99.72 ± 2.92	461.94 ± 11.58	907.42 ± 26.48	0.9927 ± 0.0029	1.2635 ± 0.0570	0.4632 ± 0.3303
Ceramide (d18:1/20:0)	0.99 ± 0.03	2.03 ± 0.14	4.96 ± 0.39	10.14 ± 0.66	55.03 ± 0.44	103.39 ± 1.24	470.29 ± 25.56	913.89 ± 18.22	0.9936 ± 0.0021	0.3367 ± 0.0178	-0.1375 ± 0.1004
Ceramide (d18:1/22:0)	1.02 ± 0.04	1.88 ± 0.10	4.96 ± 0.40	10.46 ± 0.24	54.09 ± 2.12	101.98 ± 2.46	461.64 ± 3.81	961.46 ± 18.35	0.995 ± 0.0037	0.7981 ± 0.0425	0.293 ± 0.1869
Ceramide (d18:1/24:0)	1.01 ± 0.05	1.92 ± 0.14	5.26 ± 0.27	9.68 ± 0.66	51.84 ± 0.30	102.23 ± 4.37	462.95 ± 10.71	1018.28 ± 41.09	0.9952 ± 0.0019	0.6543 ± 0.0471	0.3217 ± 0.1319
Ceramide (d18:1/24:1(15Z))	1.03 ± 0.0	1.99 ± 0.02	4.53 ± 0.17	8.79 ± 0.62	52.23 ± 0.92	104.44 ± 2.78	507.39 ± 6.56	1013.14 ± 3.03	0.9959 ± 0.0019	0.7535 ± 0.0209	-0.0465 ± 0.0531
Dihydroceramide (d18:0/12:0)	0.97 ± 0.05	2.05 ± 0.25	5.74 ± 0.79	10.88 ± 0.06	50.93 ± 1.40	99.73 ± 1.20	458.41 ± 10.15	943.11 ± 3.53	0.9931 ± 0.0002	1.0654 ± 0.0518	-0.1714 ± 0.1448
Dihydroceramide (d18:0/16:0)	0.99 ± 0.04	1.81 ± 0.43	4.8 ± 1.20	10.72 ± 0.59	49.5 ± 2.13	102.45 ± 1.30	463.59 ± 12.46	951.12 ± 19.71	0.9941 ± 0.0023	0.638 ± 0.0261	0.3016 ± 0.2113
Dihydroceramide (d18:0/18:0)	0.97 ± 0.01	2.14 ± 0.12	5 ± 0.65	10.71 ± 0.84	53.03 ± 2.45	100.61 ± 4.76	474.38 ± 15.92	921.93 ± 30.09	0.9913 ± 0.0044	0.715 ± 0.0361	0.2234 ± 0.1465
Dihydroceramide (d18:0/24:0)	1.01 ± 0.04	1.92 ± 0.11	5.15 ± 0.50	10.4 ± 0.37	52.23 ± 1.14	101.9 ± 2.97	440.73 ± 6.83	1014.62 ± 42.57	0.9938 ± 0.0004	0.3159 ± 0.0242	0.154 ± 0.0981
Dihydroceramide (d18:0/24:1(15Z))	1.03 ± 0.03	1.95 ± 0.06	4.65 ± 0.46	9.59 ± 1.22	50.4 ± 1.41	102.79 ± 3.22	503.79 ± 19.80	1058.92 ± 37.65	0.9942 ± 0.0021	0.3538 ± 0.0083	-0.133 ± 0.0696
Sphingosine (d18:1)	0.99 ± 0.05	1.97 ± 0.20	5.51 ± 0.07	10.21 ± 0.03	49.8 ± 0.64	100.81 ± 0.71	472.38 ± 6.07	967.23 ± 21.43	0.9952 ± 0.0016	0.7433 ± 0.0186	0.2422 ± 0.1187
Sphinganine (d18:0)	1.01 ± 0.04	1.92 ± 0.13	2.12 ± 0.19	10.26 ± 0.19	51.11 ± 1.10	103.17 ± 3.81	485.01 ± 9.24	953 ± 25.10	0.9973 ± 0.0012	0.6893 ± 0.0250	0.1894 ± 0.0269
SM (d18:1/16:0)	0.97 ± 0.01	1.83 ± 0.51	5.22 ± 0.18	10.91 ± 0.63	53.77 ± 3.04	101.54 ± 0.38	463.21 ± 21.91	916.56 ± 22.91	0.9939 ± 0.0031	1.0747 ± 0.0491	0.6682 ± 0.8001
SM (d18:1/18:0)	0.98 ± 0.02	2.07 ± 0.04	5.36 ± 0.88	9.78 ± 0.93	53.77 ± 2.31	102.17 ± 0.67	474.12 ± 17.33	973 ± 27.08	0.9948 ± 0.0026	0.6941 ± 0.0267	0.1724 ± 0.2309
SM (d18:1/24:0)	1.03 ± 0.02	1.87 ± 0.04	4.54 ± 0.23	10.64 ± 0.38	52.14 ± 2.67	102.81 ± 3.36	494.01 ± 17.00	1002.59 ± 12.55	0.9952 ± 0.0008	0.3135 ± 0.0148	-0.0495 ± 0.0452
SM (d18:1/24:1)	1 ± 0.05	2.06 ± 0.27	4.18 ± 1.29	9.16 ± 0.58	50.73 ± 5.74	102.06 ± 4.02	503.11 ± 9.28	1024.99 ± 26.47	0.9895 ± 0.0059	0.5216 ± 0.0159	-0.1469 ± 0.1223
Glucosyl(ß) Ceramide (d18:1/16:0)	1.02 ± 0.06	1.95 ± 0.21	4.56 ± 0.27	10.42 ± 0.82	53.44 ± 0.67	109.3 ± 0.61	481.24 ± 15.69	922.21 ± 32.83	0.9911 ± 0.0019	0.3931 ± 0.0075	0.0411 ± 0.0436
Glucosyl(ß) Ceramide (d18:1/18:0)	1.02 ± 0.07	1.89 ± 0.20	5.21 ± 0.68	10.15 ± 0.32	51.79 ± 2.83	102.49 ± 4.04	481.51 ± 21.21	957.31 ± 54.30	0.9927 ± 0.0013	0.2911 ± 0.0125	-0.0426 ± 0.0824
Glucosyl(ß) Ceramide (d18:1/18:1(9Z))	0.98 ± 0.03	2.07 ± 0.14	5.33 ± 0.10	9.29 ± 0.09	48.3 ± 0.95	99.66 ± 2.23	514.49 ± 13.70	1005.96 ± 33.13	0.9972 ± 0.0009	0.2034 ± 0.0062	-0.0608 ± 0.0631
Glucosyl(ß) Ceramide (d18:1/24:1(15Z))	0.98 ± 0.06	1.74 ± 0.04	5.47 ± 0.27	8.68 ± 1.16	47.14 ± 2.55	105.53 ± 3.93	511.59 ± 33.46	1005.07 ± 47.61	0.9916 ± 0.001	0.1591 ± 0.0051	-0.1128 ± 0.02224
Sphingosine-1-Phosphate (d18:1)	1.01 ± 0.06	1.96 ± 0.18	5.07 ± 0.50	9.81 ± 0.40	52.3 ± 1.16	100.41 ± 0.35	494.55 ± 26.66	976.5 ± 17.78	0.996 ± 0.0023	1.3884 ± 0.0401	-0.2193 ± 0.0334
Sphinganine-1-Phosphate (d18:0)	0.97 ± 0.07	2.01 ± 0.23	4.82 ± 0.42	9.14 ± 0.60	53.4 ± 1.31	98.77 ± 2.77	500.13 ± 15.87	1012.34 ± 19.99	0.9911 ± 0.0046	1.4335 ± 0.0578	-0.1652 ± 0.0496

Basit_et_al_ABC_Supplementary_Datasheet

Human naive plasma levels of Sphingolipids

Sphingolipids	Plasma Conc. (nM)
Ceramide (d18:1/12:0)	1.57 ± 2.68
Ceramide (d18:1/14:0)	below LLOQ (0.94 ± 0.47*)
Ceramide (d18:1/16:0)	66.52 ± 9.62
Ceramide (d18:1/18:0)	63.82 ± 22.53
Ceramide (d18:1/20:0)	180.84 ± 37.48
Ceramide (d18:1/22:0)	2738.37 ± 875.16
Ceramide (d18:1/24:0)	14165.92 ± 5042.28
Ceramide (d18:1/24:1(15Z))	3612.5 ± 1792.45
Dihydroceramide (d18:0/16:0)	21.3 ± 9.21
Dihydroceramide (d18:0/18:0)	52.24 ± 20.85
Dihydroceramide (d18:0/24:0)	1088.01 ± 490.85
Dihydroceramide (d18:0/24:1(15Z))	522.58 ± 172.76
Sphingosine (d18:1)	83.63 ± 57.68
Sphinganine (d18:0)	38.83 ± 20.54
Sphingosine 1-Phosphate (d18:1)	636.61 ± 100.85
Sphinganine 1-Phosphate (d18:0)	100.64 ± 21.59
HexCer (d18:1/16:0)	45.85 ± 10.11
HexCer (d18:/18:0)	4.98 ± 1.43
HexCer (d18:1/18:1)	6.5 ± 0.49
HexCer (d18:1/24:1)	566.89 ± 343.46
SM (d18:1/16:0)	3853.61 ± 1152.77
SM (d18:1/18:0)	1277.02 ± 124.10
SM (d18:1/24:0)	11719.69 ± 2234.40
SM (d18:1/24:1)	20697.98 ± 2432.61

* below LLOQ. The estimated value is reported .

Basit_et_al_ABC_Supplementary_Datasheet

Sphingolipids - MRM Transitions	Abbreviation used in the text	Retention Time (min.)	Parent Ion	Daughter Ion	Cone Voltage (V)	Collision Energy (V)
Ceramide (d18:1/12:0)	Cer (d18:1/12:0)	3.9	464.0	264.2	25	20
Ceramide (d18:1/14:0)	Cer (d18:1/14:0)	4.2	492.0	264.2	25	20
Ceramide (d18:1/16:0)	Cer (d18:1/16:0)	4.5	520.0	264.2	25	20
Ceramide (d18:1/17:0)	Cer (d18:1/17:0)	4.6	534.0	264.2	25	20
Ceramide (d18:1/18:0)	Cer (d18:1/18:0)	4.8	548.0	264.2	25	20
Ceramide (d18:1/20:0)	Cer (d18:1/20:0)	5.1	576.0	264.2	25	20
Ceramide (d18:1/22:0)	Cer (d18:1/22:0)	5.5	604.3	264.2	25	20
Ceramide (d18:1/24:0)	Cer (d18:1/24:0)	6.0	632.0	264.2	25	20
Ceramide (d18:1/24:1(15Z))	Cer (d18:1/24:1)	5.4	630.0	264.2	25	20
Dihydroceramide (d18:0/12:0)	Cer (d18:0/12:0)	4.0	484.5	466.5	25	25
Dihydroceramide (d18:0/16:0)	Cer (d18:0/16:0)	4.6	540.5	522.5	25	25
Dihydroceramide (d18:0/18:0)	Cer (d18:0/18:0)	4.9	568.5	550.5	25	25
Dihydroceramide (d18:0/24:0)	Cer (d18:0/24:0)	6.2	652.5	634.5	25	32
Dihydroceramide (d18:0/24:1(15Z))	Cer (d18:0/24:1)	5.7	650.5	632.5	25	30
Sphingosine (d17:1)	SPH (d17:1)	2.3	286.1	268.3	20	20
sphinganine (d17:0)	SPH (d17:0)	2.5	288.1	270.1	25	15
Sphingosine (d18:1)	SPH (d18:1)	2.6	300.2	282.2	20	12
Sphinganine (d18:0)	SPH (d18:0)	2.7	302.2	284.2	25	15
SM (d18:1/16:0)	SM (d18:1/16:0)	4.2	703.2	184.1	25	20
SM (d18:1/18:0)	SM (d18:1/18:0)	4.5	731.5	184.1	25	20
SM (d18:1/24:0)	SM (d18:1/24:0)	5.5	815.1	184.1	30	25
SM (d18:1/24:1)	SM (d18:1/24:1)	5.1	813.2	184.1	30	22
PC (23:0/23:0)	PC (46:0)	6.9	930.3	184.1	40	30
Glucosyl(ß) Ceramide (d18:1/12:0)	GlcCer (d18:1/12:0)	3.7	626.2	264.2	25	25
Glucosyl(ß) Ceramide (d18:1/16:0)	GlcCer (d18:1/16:0)	4.3	682.2	264.2	25	25
Glucosyl(ß) Ceramide (d18:1/18:0)	GlcCer (d18:1/18:0)	4.4	710.1	264.2	25	25
Glucosyl(ß) Ceramide (d18:1/18:1(9Z))	GlcCer (d18:1/18:1)	4.6	708.1	264.2	25	25
Glucosyl(ß) Ceramide (d18:1/24:1(15Z))	GlcCer (d18:1/24:1)	5.2	792.1	264.2	25	25
Sphingosine 1-Phosphate (d17:1)	S1P (d17:1)	2.4	366.1	250.3	25	15
sphinganine 1-Phosphate (d17:0)	S1P (d17:0)	2.6	368.2	270.3	25	15
Sphingosine 1-Phosphate (d18:1)	S1P (d18:1)	2.7	380.3	264.2	25	15
Sphinganine 1-Phosphate (d18:0)	S1P (d18:0)	2.8	382.2	284.3	25	15

Recovery in BSA and Plasma

Sphingolipids	BSA Recovery (%)	Plasma Recovery (%)	Matrix Effect (%)
Ceramide (d18:1/12:0)	91.58 ± 2.26	92.9 ± 4.87	-1.57 ± 6.69
Ceramide (d18:1/14:0)	89.97 ± 0.49	109.36 ± 6.05	-0.14 ± 5.97
Ceramide (d18:1/16:0)	88.29 ± 1.16	99.23 ± 5.62	-1.6 ± 5.31
Ceramide (d18:1/18:0)	88.54 ± 2.64	95.31 ± 8.23	-0.53 ± 6.05
Ceramide (d18:1/20:0)	86.78 ± 3.79	91.48 ± 3.56	-2.13 ± 4.44
Ceramide (d18:1/22:0)	89.08 ± 89.08	84.85 ± 1.99	2.79 ± 5.96
Ceramide (d18:1/24:0)	88.52 ± 1.21	93.18 ± 4.03	5.23 ± 5.23
Ceramide (d18:1/24:1(15Z))	94.51 ± 8.32	99.6 ± 6.17	5.52 ± 4.84
Dihydroceramide (d18:0/12:0)	92.81 ± 1.18	102.33 ± 3.92	2.67 ± 5.57
Dihydroceramide (d18:0/16:0)	91.79 ± 7.38	95.09 ± 10.37	-1.3 ± 1.92
Dihydroceramide (d18:0/18:0)	88.28 ± 2.06	90.08 ± 4.88	2.23 ± 3.89
Dihydroceramide (d18:0/24:0)	91.82 ± 8.83	95.57 ± 3.16	-1.19 ± 5.56
Dihydroceramide (d18:0/24:1(15Z))	92.96 ± 7.17	101.37 ± 8.25	6.4 ± 4.59
Sphingosine (d18:1)	116.11 ± 2.94	71.93 ± 4.19	0.8 ± 4.04
Sphinganine (d18:0)	116.86 ± 1.16	93.69 ± 4.90	-3.53 ± 5.24
SM (d18:1/16:0)	81.99 ± 2.11	98.36 ± 0.95	-8.96 ± 5.21
SM (d18:1/18:0)	83.28 ± 11.97	98.58 ± 0.80	-9.1 ± 4.47
SM (d18:1/24:0)	83.85 ± 6.71	95.48 ± 2.71	2.27 ± 1.50
SM (d18:1/24:1)	82.75 ± 6.72	98.86 ± 2.43	-2.29 ± 2.15
Glucosyl(ß) Ceramide (d18:1/16:0)	88.19 ± 2.76	95.37 ± 8.41	-3.58 ± 3.44
Glucosyl(ß) Ceramide (d18:1/18:0)	87.81 ± 3.87	93.71 ± 7.85	-3.67 ± 1.80
Glucosyl(ß) Ceramide (d18:1/18:1(9Z))	93.33 ± 4.92	111.78 ± 1.23	-5.75 ± 6.57
Glucosyl(ß) Ceramide (d18:1/24:1(15Z))	90.39 ± 2.78	102.87 ± 9.57	-2.36 ± 3.73
Sphingosine-1-Phosphate (d18:1)	76.93 ± 2.45	105.01 ± 14.08	-6.76 ± 1.85
Sphinganine-1-Phosphate (d18:0)	75.89 ± 0.71	86.64 ± 4.21	-8.27 ± 0.91

Precision and Accuracy in BSA

Sphingolipids	Intra assay				Inter assay			
	%CV		% Accuracy		%CV		% Accuracy	
	Mean	S.D.	Mean	S.D.	Mean	S.D.	Mean	S.D.
Ceramide (d18:1/12:0)	5.11	2.82	96.93	1.63	5.85	1.34	97.26	1.37
Ceramide (d18:1/14:0)	4.10	0.65	93.12	3.07	3.35	0.60	97.82	4.09
Ceramide (d18:1/16:0)	4.85	1.86	97.87	1.77	2.78	1.13	96.13	3.46
Ceramide (d18:1/18:0)	5.10	2.42	96.00	2.92	1.58	1.04	94.78	2.21
Ceramide (d18:1/20:0)	6.10	4.22	97.74	3.37	0.95	0.44	97.52	4.08
Ceramide (d18:1/22:0)	4.97	2.95	96.47	0.86	3.68	0.51	97.09	2.63
Ceramide (d18:1/24:0)	6.07	5.35	102.17	1.28	6.58	1.24	98.20	2.42
Ceramide (d18:1/24:1(15Z))	4.75	3.49	101.63	1.53	3.74	1.90	101.96	3.45
Dihydroceramide (d18:0/12:0)	4.68	1.49	97.05	4.80	3.08	1.61	96.08	3.83
Dihydroceramide (d18:0/16:0)	4.18	1.78	96.39	0.93	3.08	1.82	96.13	2.12
Dihydroceramide (d18:0/18:0)	6.56	4.03	96.80	2.28	3.19	1.52	95.79	1.52
Dihydroceramide (d18:0/24:0)	6.19	4.93	101.76	0.20	6.94	1.86	98.81	2.23
Dihydroceramide (d18:0/24:1(15Z))	2.80	2.67	98.43	1.63	5.10	1.16	100.66	4.00
Sphingosine (d18:1)	6.15	3.51	95.20	1.65	1.91	0.95	94.72	1.53
Sphinganine (d18:0)	5.28	0.95	94.49	3.89	2.73	0.85	95.09	2.26
SM (d18:1/16:0)	5.60	2.72	96.54	5.62	5.68	2.03	96.70	3.13
SM (d18:1/18:0)	5.25	3.17	95.46	2.08	6.53	1.48	97.61	1.44
SM (d18:1/24:0)	7.94	3.21	99.10	1.85	6.03	0.59	97.56	2.32
SM (d18:1/24:1)	5.50	2.22	98.07	4.09	4.30	1.99	100.80	4.39
Glucosyl(ß) Ceramide (d18:1/16:0)	2.76	0.41	101.05	3.94	3.09	0.44	99.48	4.40
Glucosyl(ß) Ceramide (d18:1/18:0)	2.96	0.99	97.07	5.03	3.68	2.34	97.28	3.81
Glucosyl(ß) Ceramide (d18:1/18:1(9Z))	4.82	4.12	102.59	3.08	4.60	3.71	101.23	2.10
Glucosyl(ß) Ceramide (d18:1/24:1(15Z))	4.63	2.90	99.11	1.73	4.26	2.05	97.42	3.88
Sphingosine-1-Phosphate (d18:1)	4.12	4.64	98.55	1.12	3.37	1.04	99.42	2.49
Sphinganine-1-Phosphate (d18:0)	4.19	0.24	103.08	5.03	2.79	0.74	101.11	3.17

Basit et al_ABC_SuppFile_1_Matrix Effect Evaluation

Aim of the Experiment

Estimate the matrix effect on the quantification of endogeous sphignolipids from human plasma.

Experimental details

Matrix effect was evaluated using the post-column infusion method*: a mixture of authentic standards of analytes diluted in 9:1 MeOH/CHCl3 to a final 10μM concentration was infused post-column in the LC-MS/MS system using a tee union. Repeated injections of extracted human plasma samples were performed with the aim to investigate significant decreases or increases in the analyte MRM ion currents.

Results

Most metabolites targeted in the present paper do not suffer from any major matrix effect from human plasma. Five metabolites suffer from substantial matrix effects and would perhaps benefit from the use of deuterated internal standards.

*Geis-Asteggiante L. et al. (2012) Ruggedness testing and validation of a practical analytical method for >100 veterinary drug residues in bovine muscle by ultrahigh performance liquid chromatography-tandem mass spectrometry. J Chromatogr A 1258:43-54.

No significant Matrix Effect

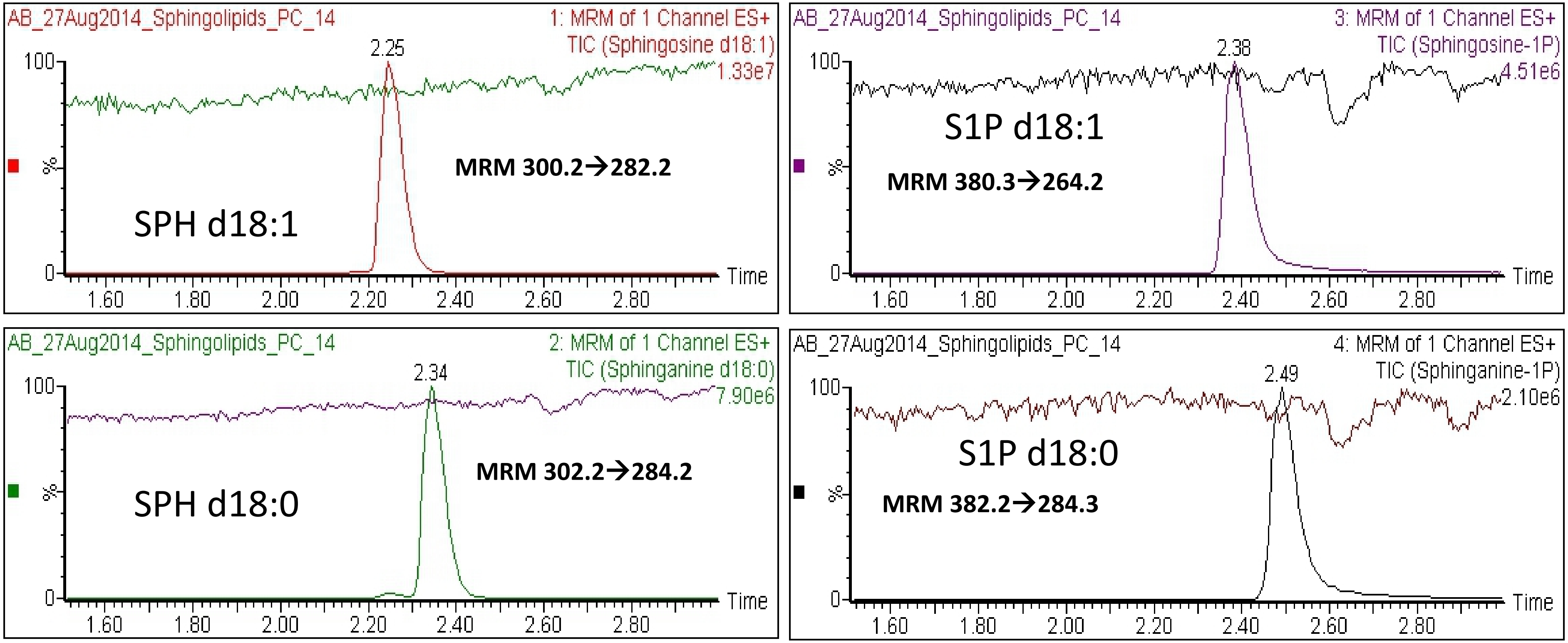

No significant Matrix Effect

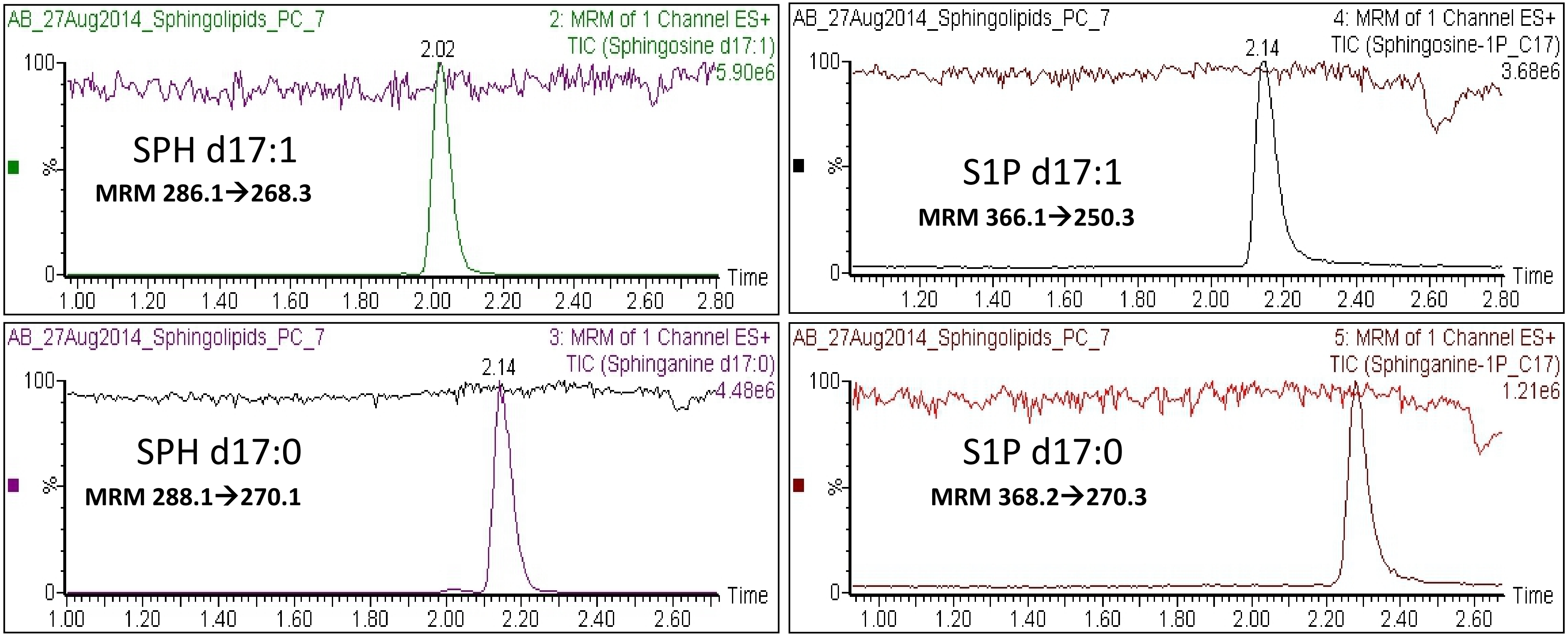

No significant Matrix Effect

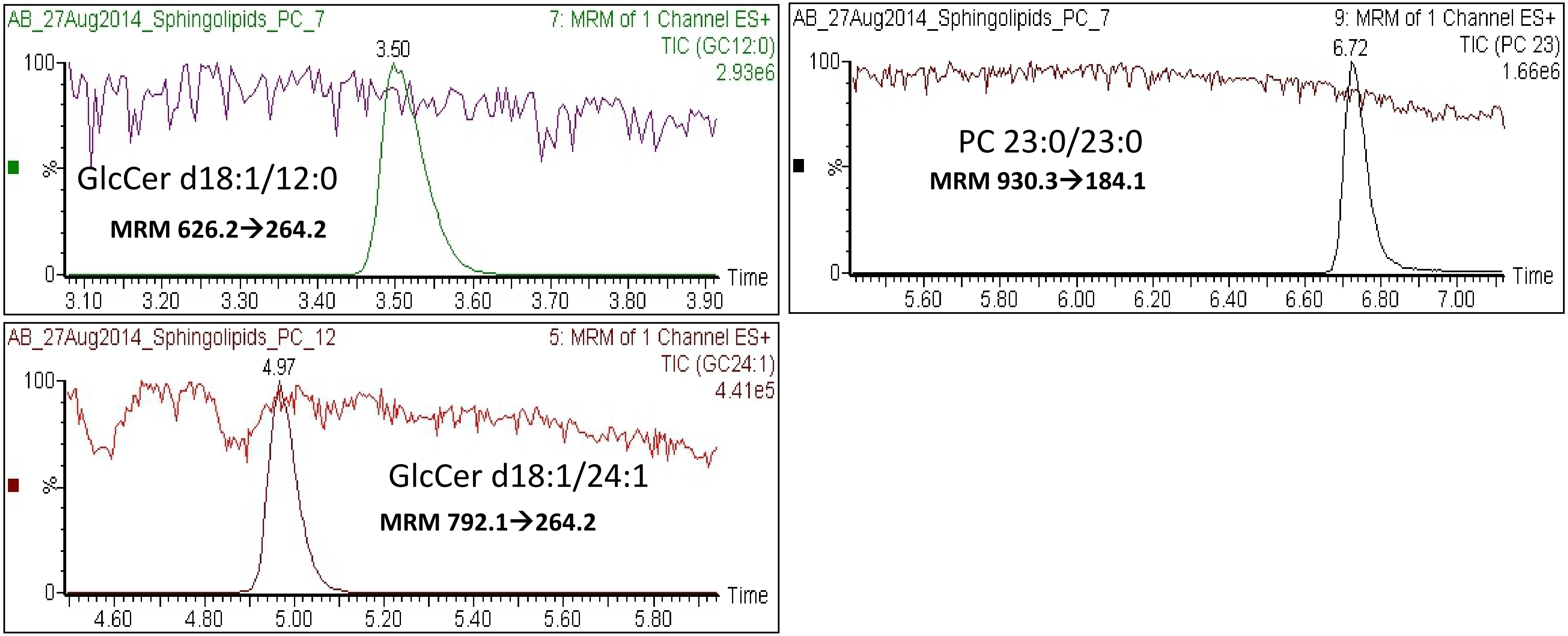

No significant Matrix Effect
Increase in signal is due to highly abundant SM present in plasma

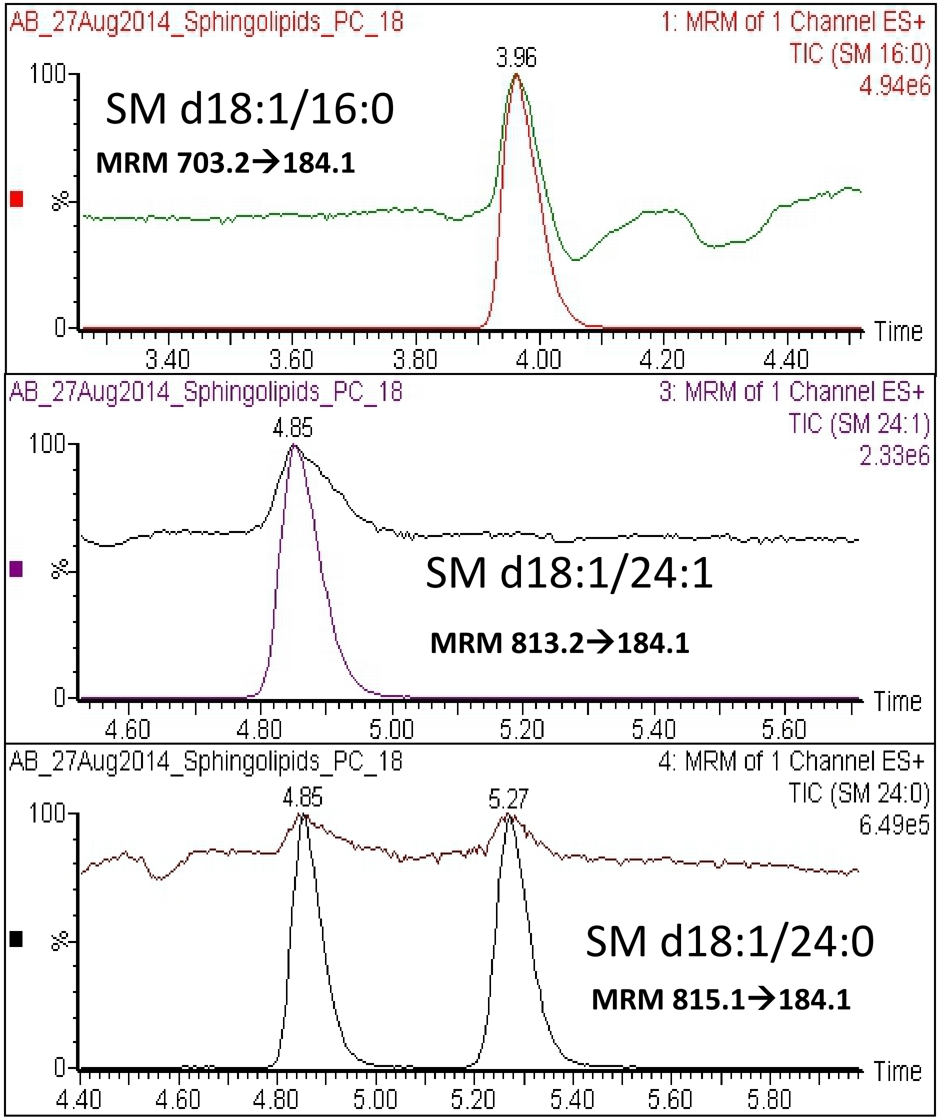

No significant Matrix Effect

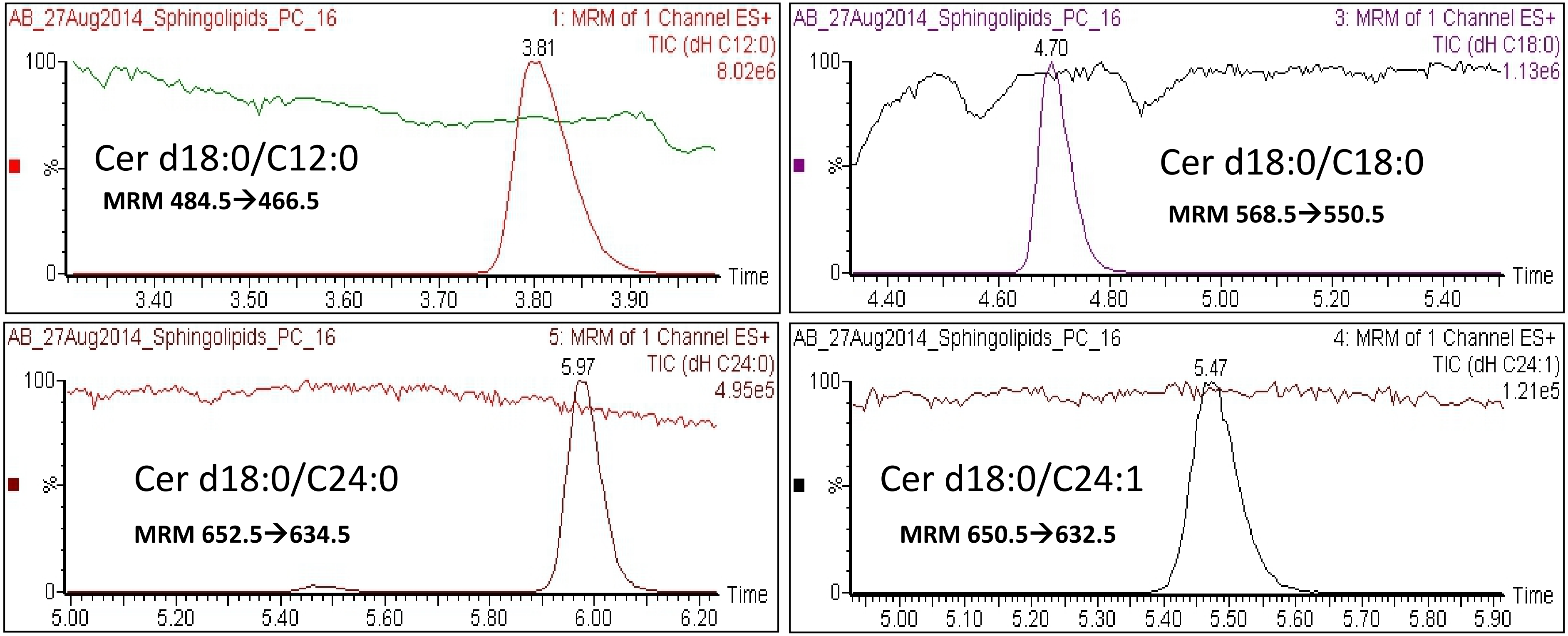

No significant Matrix Effect

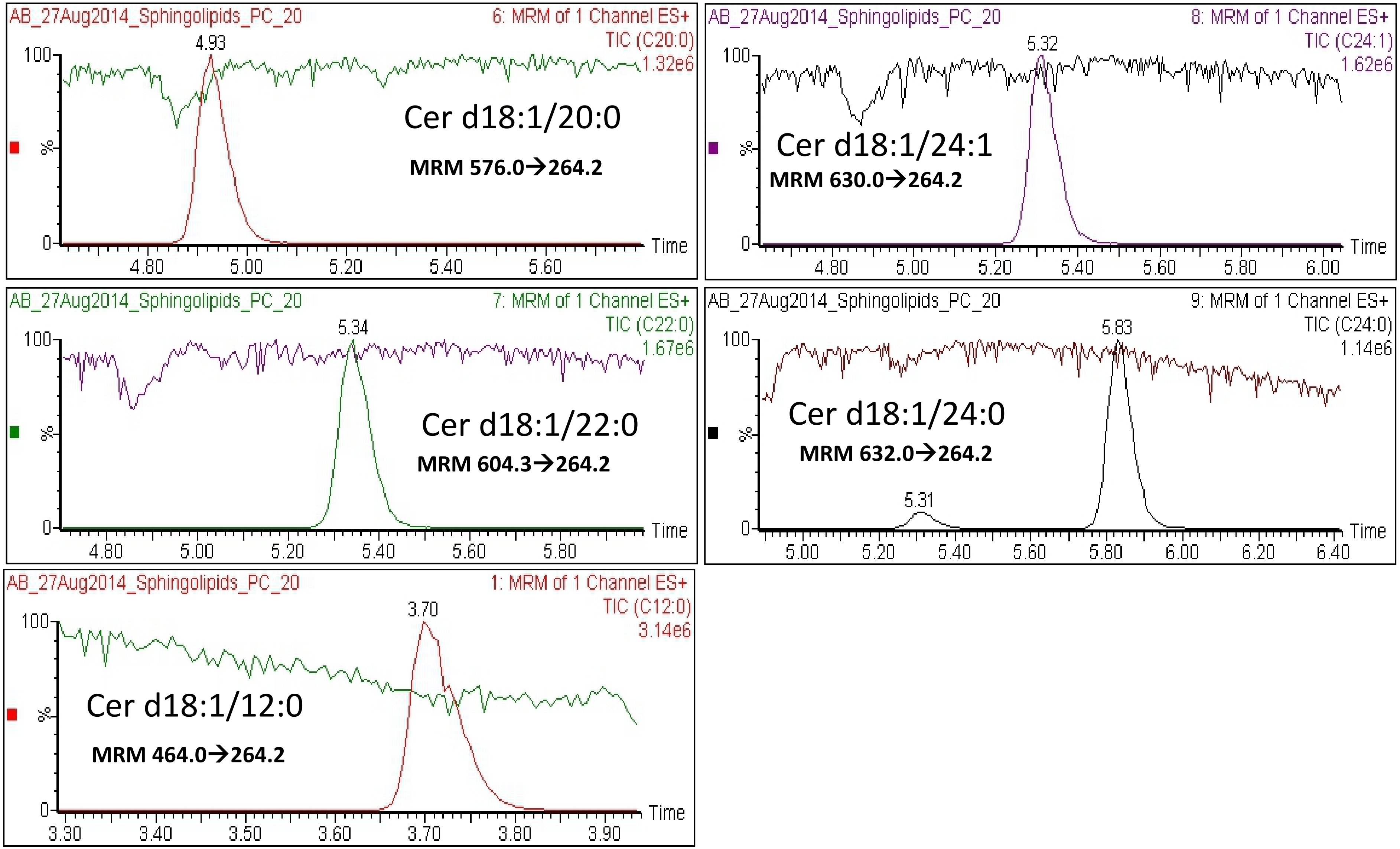

Minor Matrix Effect (<20%)

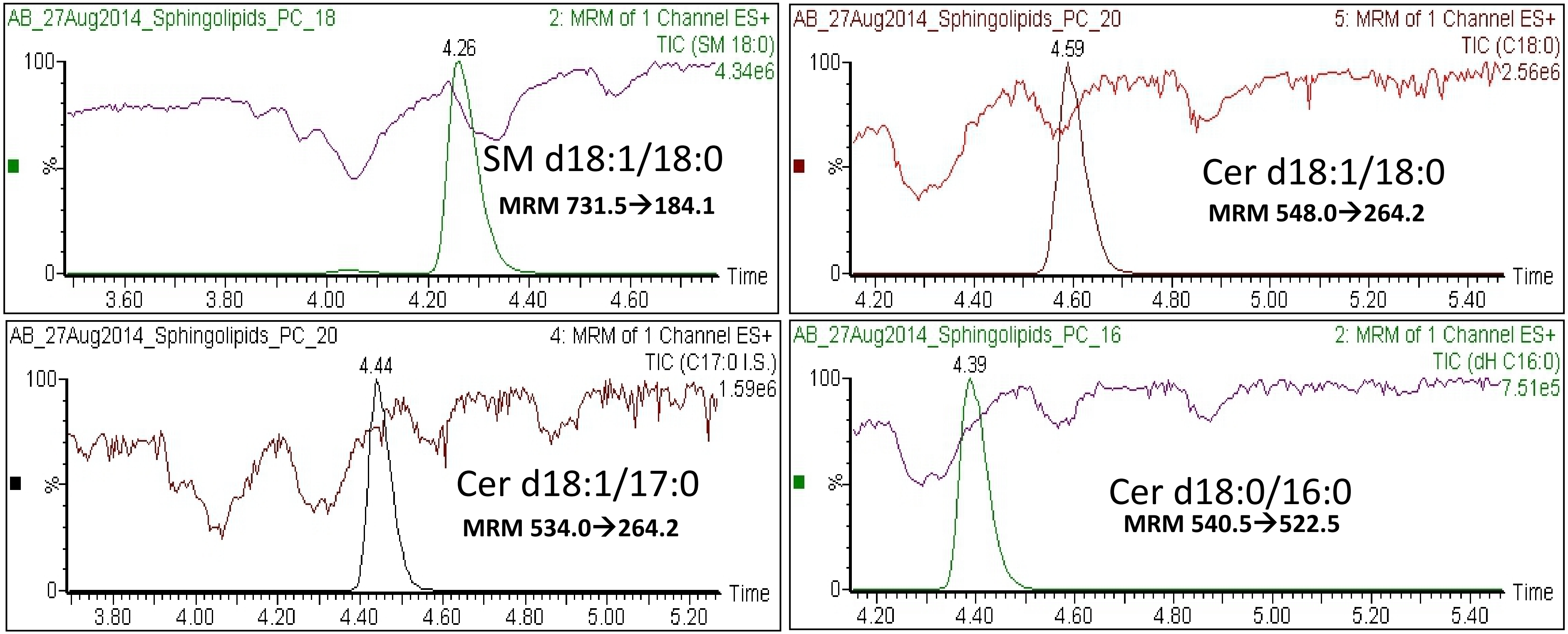

Major Matrix Effect (>20%)

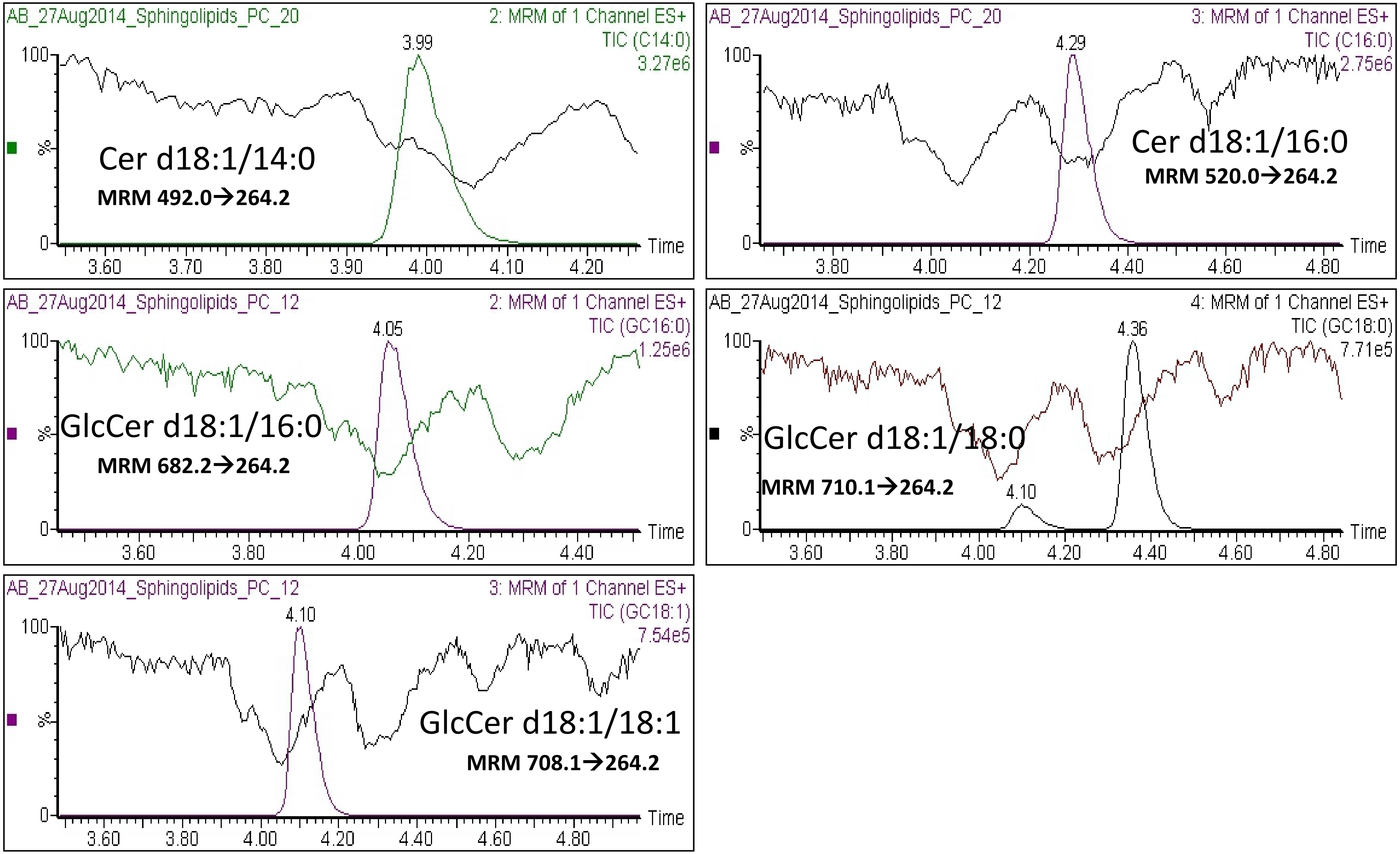

Basit et al_ABC_SuppFile_2_SM_PC_Interference_Test

Aim of the Experiment

To show that no potentially interfering phosphatylcholines (PC) from human plasma are coeluting with the four sphingomyelins (SM) targeted by our method

Experimental details

The LC-MS/MS method reported in the paper was transferred to a UPLC-Qtof system (Waters G2). Sphingomyelin standards and human plasma were prepared as described in the paper and injected (separately) in the system. High resolution mass spectra at the retention time of eluting SM (± 10 seconds) were acquired in ESI+ mode in the 50-1200 m/z range for both standards and extracted plasma. Spectra were recalibrated in real-time by infusing Leucine Enkephalin (2ng/ml) as reference mass. MS spectra of standards and extracted plasma were compared with the expected isotopic profile of the corresponding SM. A brute formula was calculated from the accurate mass of the extracted plasma, setting a mass tolerance of 10ppm and allowing the following options: **C**: 0 to 100, **H**: 0 to 500, **N**: 0 to 5, **O**: 0 to 10, **P**: 0 to 1. The accurate mass recorded from extracted plasma was then searched against Lipidmaps* for phosphatidylcholines precursor ion search, setting a m/z tolerance of 0,5m/z and allowing [M+H]+, [M+K]+, [M+Na]+ and [M+NH4]+ as adducts and limiting the search to PC headgroup. The m/z tolerance was set considering the bandwidth selection of the first quadrupole of the Xevo triple quad MS used for SM quantification.

Results

The brute formula of the SM was always correctly guessed by the software (red arrow), but the corresponding m/z value, when searched against Lipidmaps never returned a PC with a compatible brute formula. Furthemore, all reported PCs are ammonium adducts, which is incompatible with the usual PC detection as [M+H]+ species.

*(http://www.lipidmaps.org/tools/ms/glycerophospholipids_batch_bulk.html)

SM d18:1/16:0)   Brute Formula C39H79N2O6P

High Resolution LC-MS traces

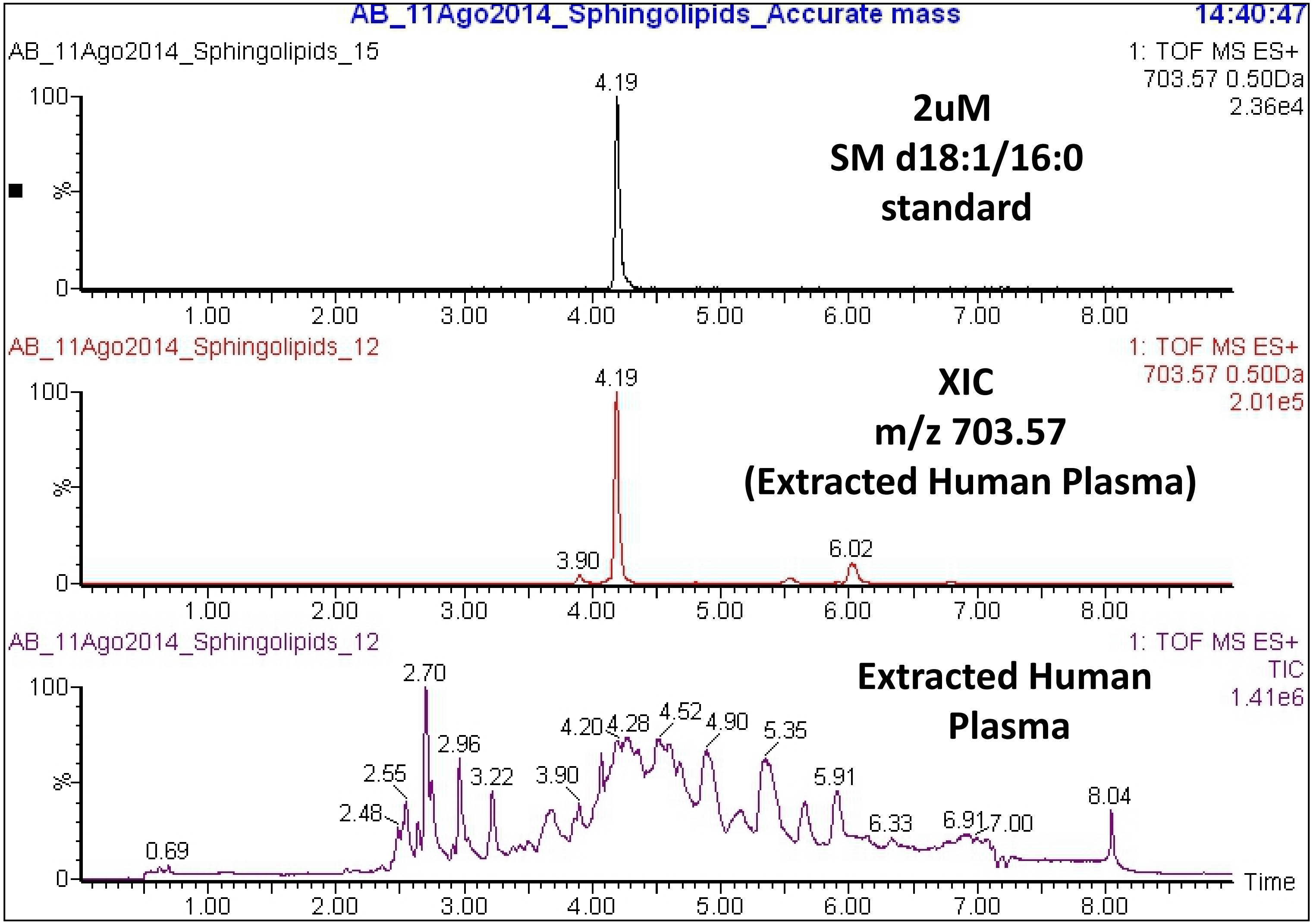

High Resolution Mass Spectra At 4,15 to 4,25 min. RT

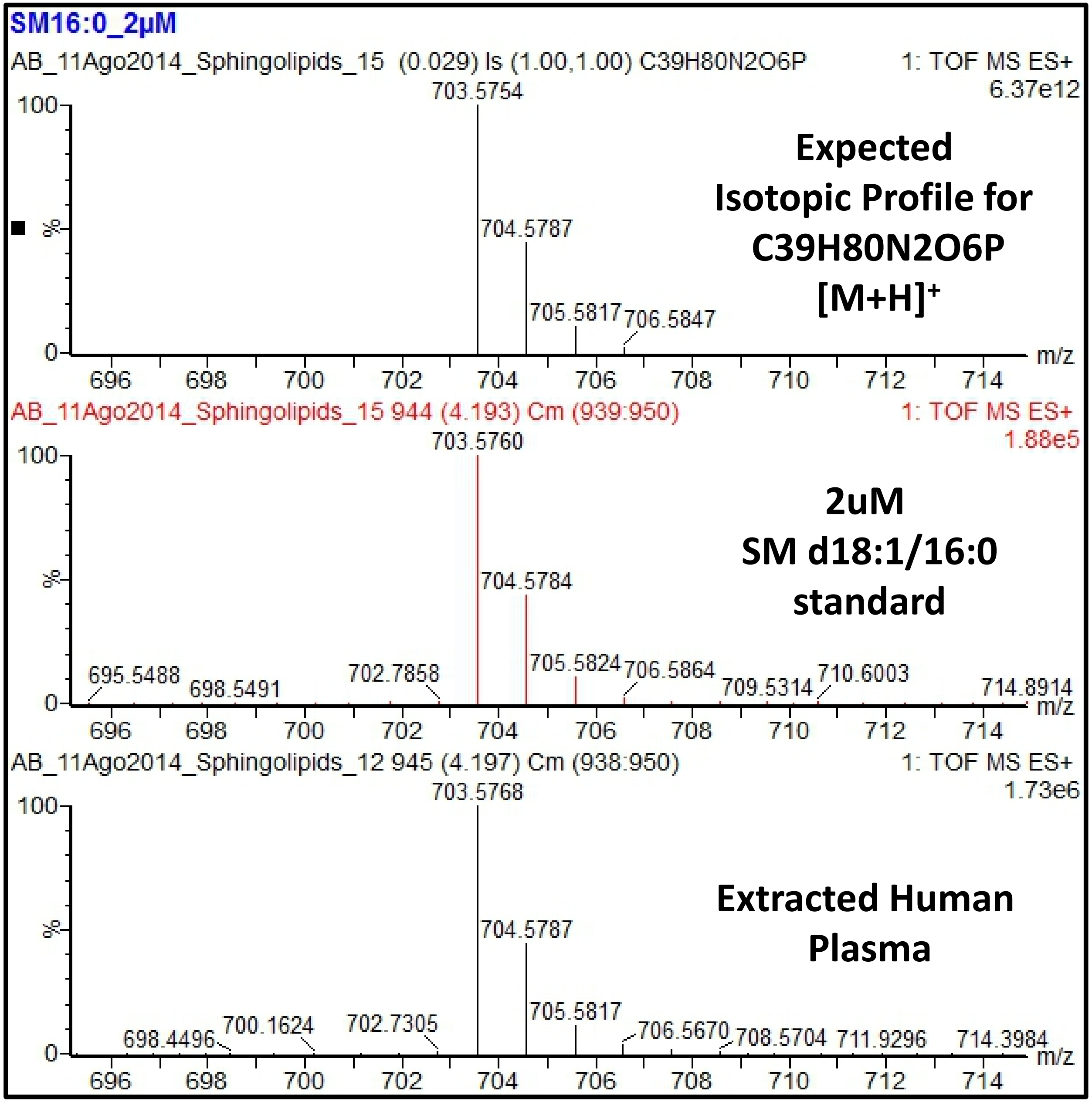

Mass	Calc. Mass	mDa	PPM	DBE	Formula	i-FIT	i-FIT Norm	Fit Conf %	C	H	N	O	P
703.5768	703.5778	-1.0	-1.4	10.5	C46 H75 N2 O3	31.2	10.958	0.00	46	75	2	3	
	703.5754	1.4	2.0	1.5	C39 H80 N2 O6 P	22.8	2.619	7.29	39	80	2	6	1
	703.5794	-2.6	-3.7	5.5	C44 H80 O4 P	29.7	9.491	0.01	44	80		4	1
	703.5737	3.1	4.4	6.5	C41 H75 N4 O5	26.5	6.260	0.19	41	75	4	5	
	703.5808	-4.0	-5.7	10.5	C45 H76 N4 P	31.5	11.226	0.00	45	76	4		1
	703.5724	4.4	6.3	1.5	C40 H79 O9	22.7	2.460	8.54	40	79		9	
	703.5818	-5.0	-7.1	14.5	C51 H75 O	34.8	14.580	0.00	51	75		1	
	703.5836	-6.8	-9.7	1.5	C39 H79 N2 O8	20.4	0.175	83.97	39	79	2	8	

Elemental composition analysis for 703,5768 m/z (+/- 10ppm) detected in blank plasma

LIPID Metabolites and Pathways Strategy (LIPID MAPS)

Lipidomics Gateway

Search ?

Home | Lipidomics Update | Resources | Meetings | Tutorials | Protocols | About

Overview | Publications | Classification | Standards | Data | Databases | Pathways | Tools | Services | Links | Downloads

Possible Glycerophospholipid Bulk Structures

Input Mass	Matched Mass	Delta	Abbreviation	Formula	Ion
703.5768	703.5021	.0747	PC(29:3)	$C_{37}H_{68}NO_8P$	[M+NH4]+
703.5768	703.5384	.0384	PC(P-30:2)	$C_{38}H_{72}NO_7P$	[M+NH4]+
703.5768	703.5384	.0384	LPC(30:3)	$C_{38}H_{72}NO_7P$	[M+NH4]+
703.5768	703.5384	.0384	PC(O-30:3)	$C_{38}H_{72}NO_7P$	[M+NH4]+

LipidMaps Database Search for 703,5768 m/z detected in blank plasma
Search Tolerance set to +/- 0,5 m/z units

SM d18:1/18:0   Brute Formula C41H83N2O6P

High Resolution LC-MS traces

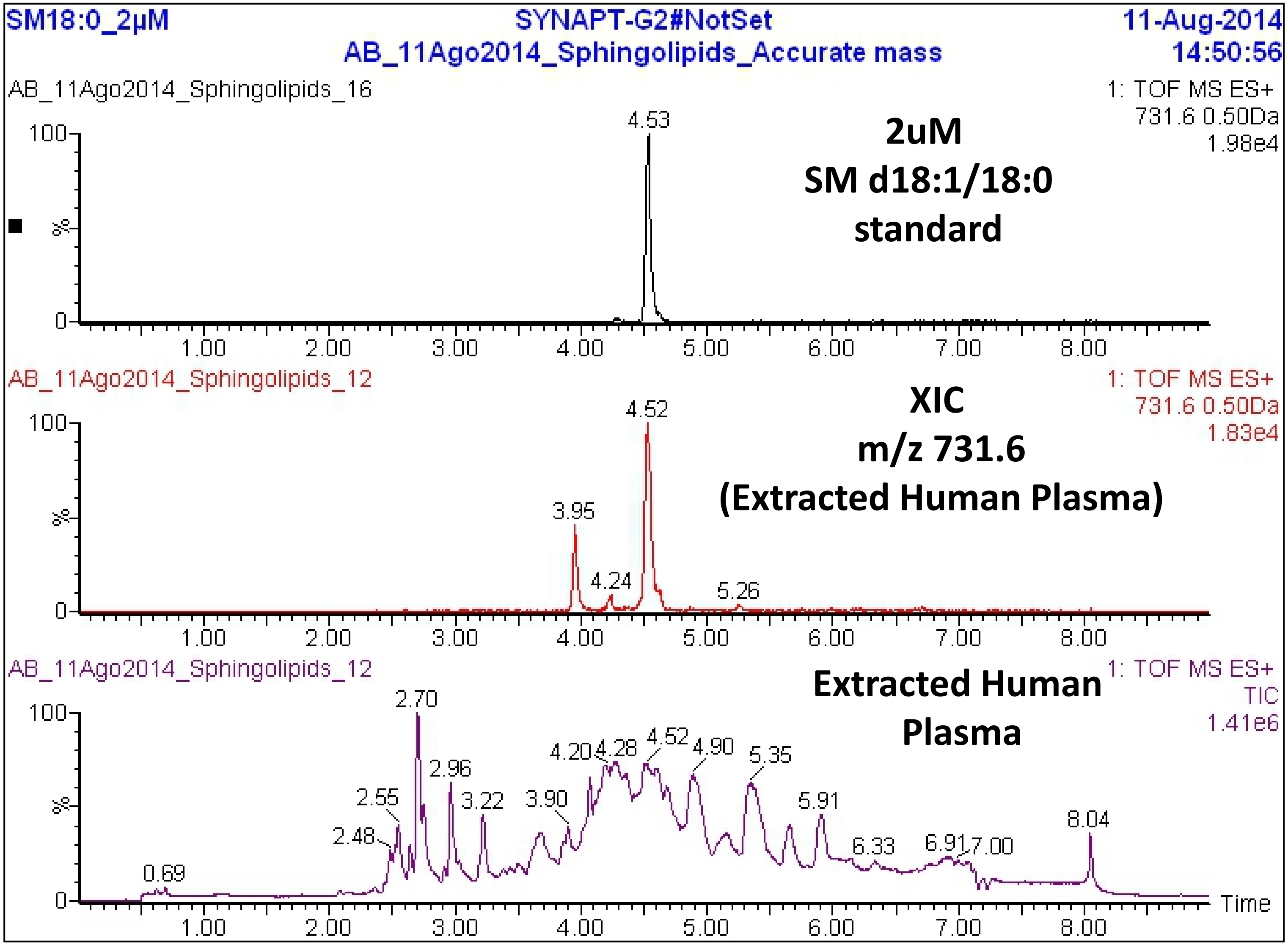

High Resolution Mass Spectra At 4,40 to 4,60 min. RT

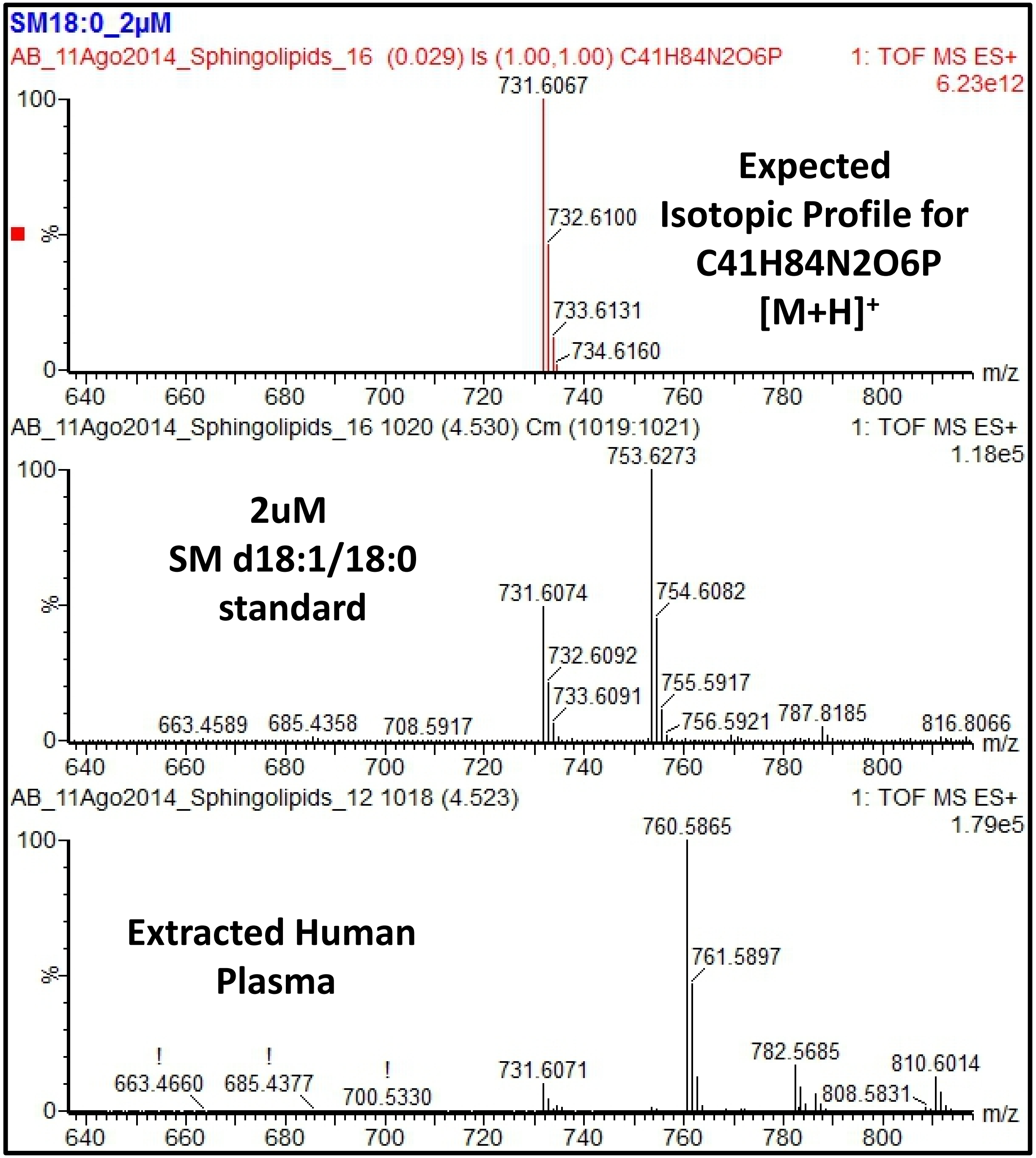

Mass	Calc. Mass	mDa	PPM	DBE	Formula	i-FIT	i-FIT Norm	Fit Conf %	C	H	N	O	P
731.6071	731.6067	0.4	0.5	1.5	C41 H84 N2 O6 P	150.8	0.488	61.39	41	84	2	6	1
	731.6091	-2.0	-2.7	10.5	C48 H79 N2 O3	153.9	3.516	2.97	48	79	2	3	
	731.6050	2.1	2.9	6.5	C43 H79 N4 O5	152.6	2.265	10.38	43	79	4	5	
	731.6037	3.4	4.6	1.5	C42 H83 O9	152.3	1.929	14.52	42	83		9	
	731.6107	-3.6	-4.9	5.5	C46 H84 O4 P	153.1	2.746	6.42	46	84		4	1
	731.6121	-5.0	-6.8	10.5	C47 H80 N4 P	154.0	3.619	2.68	47	80	4		1
	731.6131	-6.0	-8.2	14.5	C53 H79 O	155.7	5.384	0.46	53	79		1	
	731.6008	6.3	8.6	10.5	C48 H80 N2 O P	154.8	4.448	1.17	48	80	2	1	1

Elemental composition analysis for 731,6071 m/z (+/- 10ppm) detected in blank plasma.

LIPID Metabolites and Pathways Strategy (LIPID MAPS)

Lipidomics Gateway

Search ?

Home | Lipidomics Update | Resources | Meetings | Tutorials | Protocols | About

Overview | Publications | Classification | Standards | Data | Databases | Pathways | Tools | Services | Links | Downloads

Possible Glycerophospholipid Bulk Structures

Input Mass	Matched Mass	Delta	Abbreviation	Formula	Ion
731.6071	731.5334	.0737	PC(31:3)	$C_{39}H_{72}NO_8P$	[M+NH4]+
731.6071	731.5697	.0374	LPC(32:3)	$C_{40}H_{76}NO_7P$	[M+NH4]+
731.6071	731.5697	.0374	PC(O-32:3)	$C_{40}H_{76}NO_7P$	[M+NH4]+
731.6071	731.5697	.0374	PC(P-32:2)	$C_{40}H_{76}NO_7P$	[M+NH4]+

LipidMaps Database Search for 731,6071 m/z detected in blank plasma
Search Tolerance set to +/- 0,5 m/z units

SM d18:1/24:1   Brute Formula C47H93N2O6P

High Resolution LC-MS traces

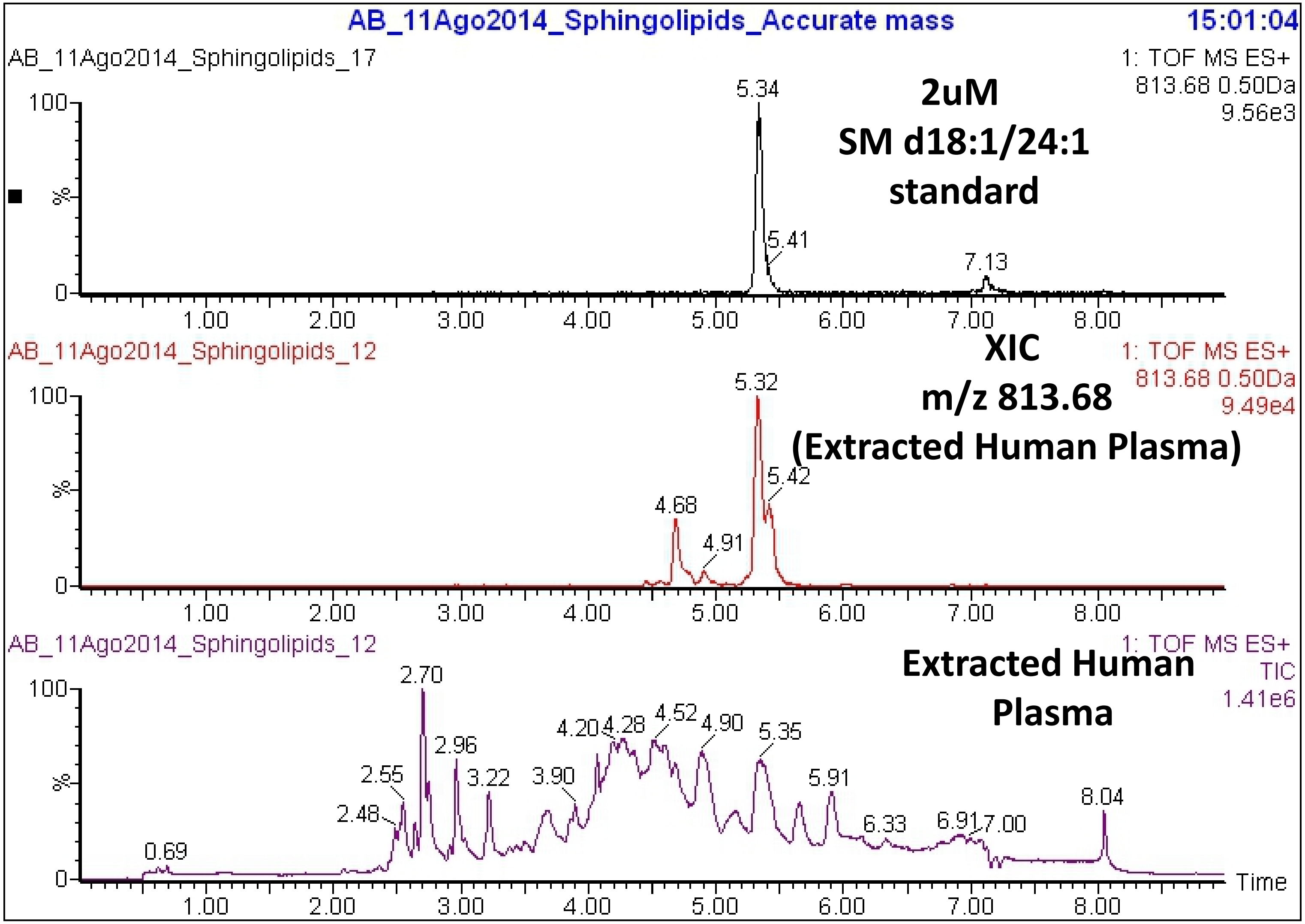

High Resolution Mass Spectra
At 5,20 to 5,40 min. RT

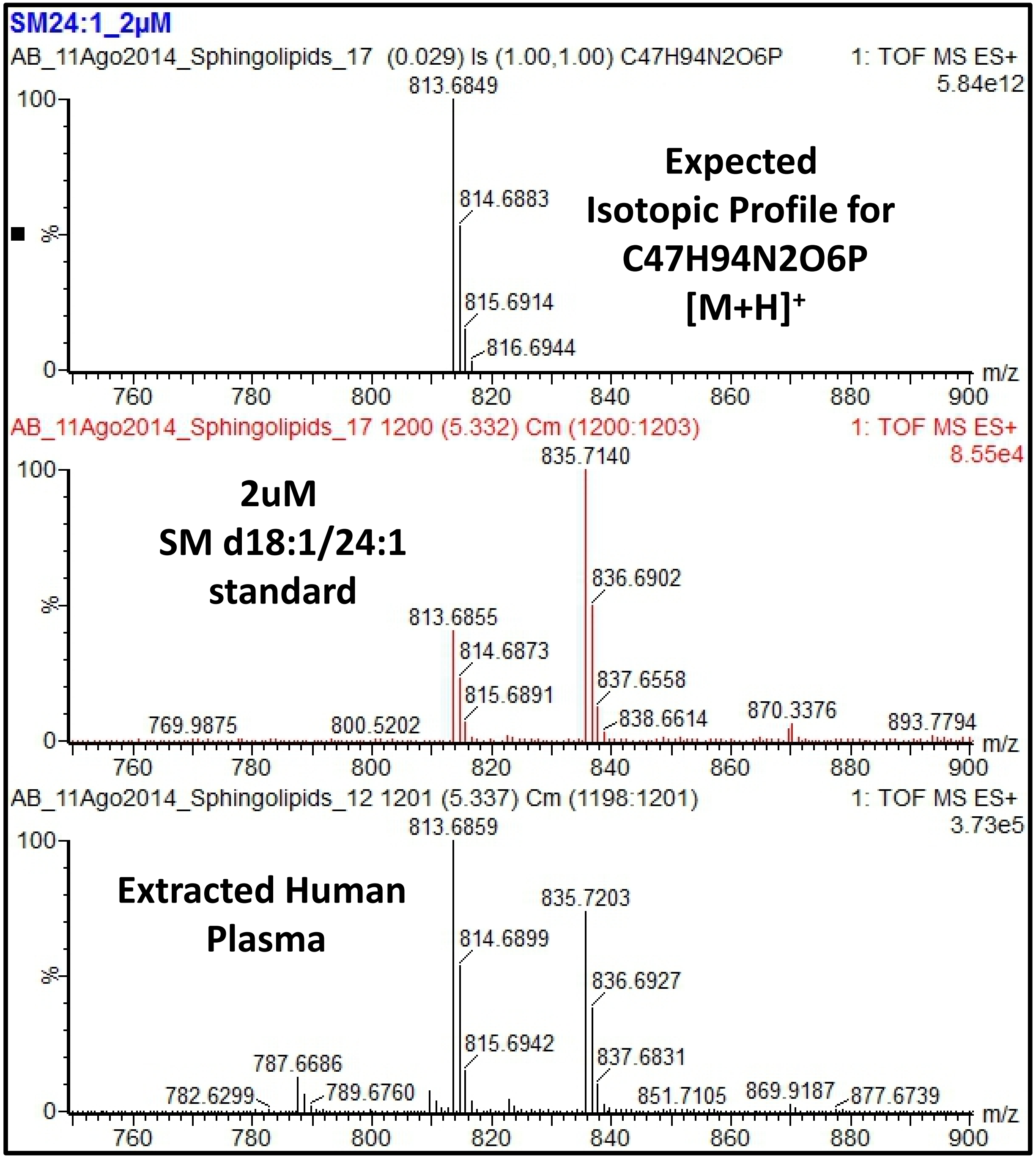

Mass	Calc. Mass	mDa	PPM	DBE	Formula	i-FIT	i-FIT Norm	Fit Conf %	C	H	N	O	P
813.6859	813.6850	0.9	1.1	2.5	C47 H94 N2 O6 P	23.0	0.001	99.90	47	94	2	6	1
	813.6873	-1.4	-1.7	11.5	C54 H89 N2 O3	35.5	12.431	0.00	54	89	2	3	
	813.6833	2.6	3.2	7.5	C49 H89 N4 O5	32.2	9.167	0.01	49	89	4	5	
	813.6890	-3.1	-3.8	6.5	C52 H94 O4 P	33.9	10.850	0.00	52	94		4	1
	813.6892	-3.3	-4.1	-1.5	C42 H93 N4 O10	34.5	11.511	0.00	42	93	4	10	
	813.6820	3.9	4.8	2.5	C48 H93 O9	30.1	7.080	0.08	48	93		9	
	813.6903	-4.4	-5.4	11.5	C53 H90 N4 P	35.9	12.833	0.00	53	90	4		1
	813.6809	5.0	6.1	-1.5	C42 H94 N4 O8 P	36.1	13.049	0.00	42	94	4	8	1
	813.6913	-5.4	-6.6	15.5	C59 H89 O	38.9	15.907	0.00	59	89		1	
	813.6791	6.8	8.4	11.5	C54 H90 N2 O P	37.8	14.782	0.00	54	90	2	1	1

Elemental composition analysis for 813,6859 m/z (+/- 10ppm) detected in blank plasma.

LIPID Metabolites and Pathways Strategy (LIPID MAPS)

Lipidomics Gateway

Search ?

Home | Lipidomics Update | Resources | Meetings | Tutorials | Protocols | About

Overview | Publications | Classification | Standards | Data | Databases | Pathways | Tools | Services | Links | Downloads

Possible Glycerophospholipid Bulk Structures

Input Mass	Matched Mass	Delta	Abbreviation	Formula	Ion
813.6859	813.6116	.0743	PC(37:4)	$C_{45}H_{82}NO_8P$	[M+NH4]+
813.6859	813.6480	.0379	PC(O-38:4)	$C_{46}H_{86}NO_7P$	[M+NH4]+
813.6859	813.6480	.0379	PC(P-38:3)	$C_{46}H_{86}NO_7P$	[M+NH4]+

LipidMaps Database Search for 813,6859 m/z detected in blank plasma
Search Tolerance set to +/- 0,5 m/z units

SM d18:1/24:0   Brute Formula C47H95N2O6P

High Resolution LC-MS traces

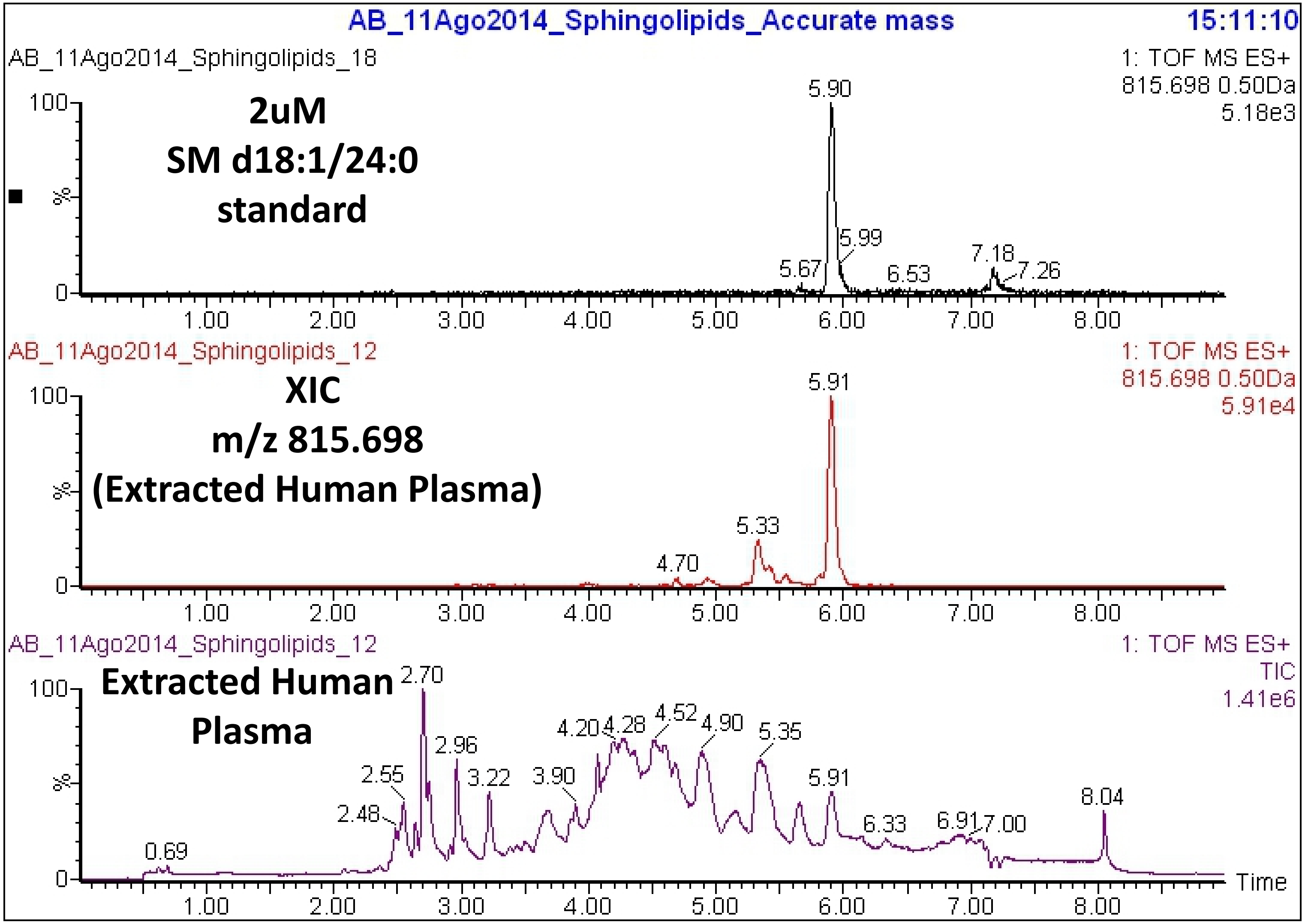

High Resolution Mass Spectra At 5,20 to 5,40 min. RT

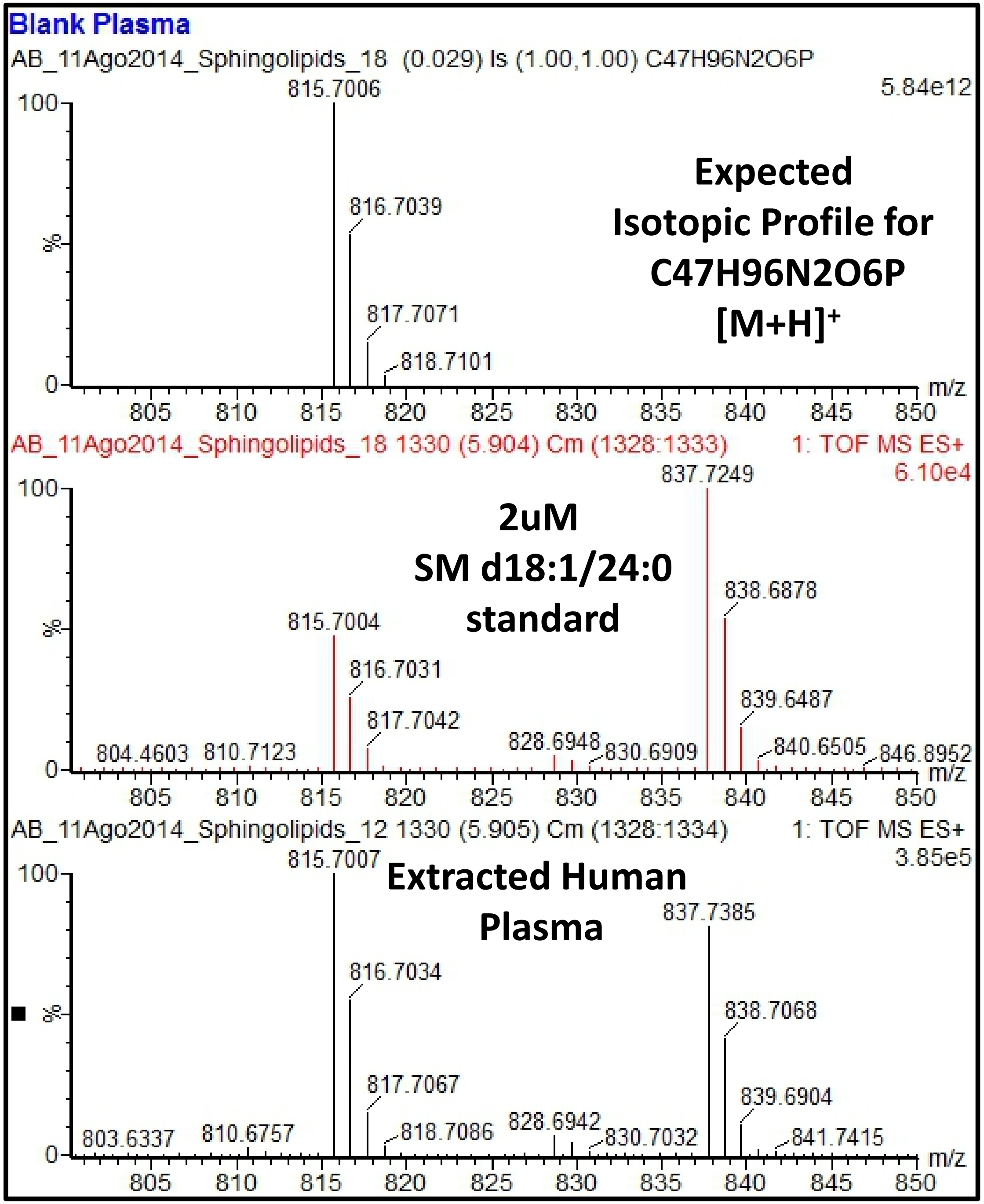

Mass	Calc. Mass	mDa	PPM	DBE	Formula	i-FIT	i-FIT Norm	Fit Conf %	C	H	N	O	P
815.7007	815.7006	0.1	0.1	1.5	C47 H96 N2 O6 P	17.6	0.234	79.13	47	96	2	6	1
	815.6989	1.8	2.2	6.5	C49 H91 N4 O5	19.8	2.477	8.40	49	91	4	5	
	815.7030	-2.3	-2.8	10.5	C54 H91 N2 O3	24.8	7.453	0.06	54	91	2	3	
	815.6976	3.1	3.8	1.5	C48 H95 O9	19.6	2.273	10.30	48	95		9	
	815.7046	-3.9	-4.8	5.5	C52 H96 O4 P	23.4	6.048	0.24	52	96		4	1
	815.7060	-5.3	-6.5	10.5	C53 H92 N4 P	25.1	7.772	0.04	53	92	4		1
	815.6947	6.0	7.4	10.5	C54 H92 N2 O P	25.5	8.091	0.03	54	92	2	1	1
	815.7070	-6.3	-7.7	14.5	C59 H91 O	28.6	11.237	0.00	59	91		1	
	815.6931	7.6	9.3	15.5	C56 H87 N4	27.6	10.196	0.00	56	87	4		
	815.7088	-8.1	-9.9	1.5	C47 H95 N2 O8	21.4	4.016	1.80	47	95	2	8	

Elemental composition analysis for 815,7007 m/z (+/- 10ppm) detected in blank plasma.

LIPID Metabolites and Pathways Strategy (LIPID MAPS)

Lipidomics Gateway

Search ?

Home | Lipidomics Update | Resources | Meetings | Tutorials | Protocols | About

Overview | Publications | Classification | Standards | Data | Databases | Pathways | Tools | Services | Links | Downloads

Possible Glycerophospholipid Bulk Structures

Input Mass	Matched Mass	Delta	Abbreviation	Formula	Ion
815.7007	815.6273	.0734	PC(37:3)	$C_{45}H_{84}NO_8P$	[M+NH4]+
815.7007	815.6636	.0371	PC(O-38:3)	$C_{46}H_{88}NO_7P$	[M+NH4]+
815.7007	815.6636	.0371	PC(P-38:2)	$C_{46}H_{88}NO_7P$	[M+NH4]+

LipidMaps Database Search for 815,7007 m/z detected in blank plasma
Search Tolerance set to +/- 0,5 m/z units
